# Control over Charge Density by Tuning the Polyelectrolyte Type and Monomer Ratio in Saloplastic-Based Ion-Exchange Membranes

**DOI:** 10.1021/acs.langmuir.3c00497

**Published:** 2023-05-01

**Authors:** Ameya Krishna B, Wiebe M. de Vos, Saskia Lindhoud

**Affiliations:** † Membrane Surface Science, Membrane Science and Technology, MESA+ Institute of Nanotechnology, University of Twente, Enschede, Overijssel 7500 AE, The Netherlands; ‡ Department of Molecules and Materials, University of Twente, Enschede, Overijssel 7500 AE, The Netherlands

## Abstract

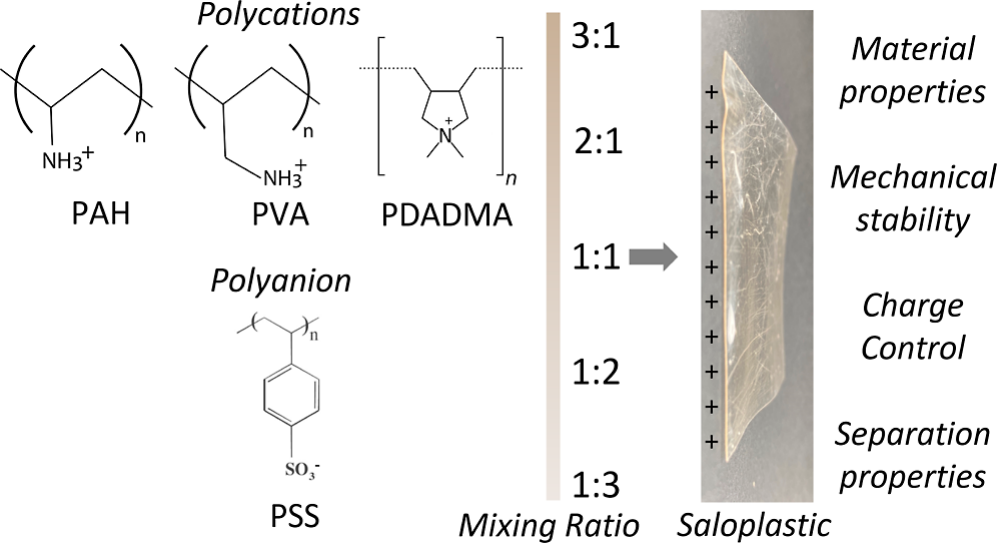

Membranes based on polyelectrolyte complexes (PECs) can now be prepared through several sustainable, organic solvent-free approaches. A recently developed approach allows PECs made by stoichiometric mixing of polyelectrolytes to be hot-pressed into dense saloplastics, which then function as ion-exchange membranes. An important advantage of PECs is that tuning their properties can provide significant control over the properties of the fabricated materials, and thus over their separation properties. This work studies the effects of two key parameters—(a) ratio of mixing and (b) choice of polyelectrolytes—on the mechanical, material, and separation properties of their corresponding hot-pressed saloplastic-based ion-exchange membranes. By varying these two main parameters, charge density—the key property of any IEM—was found to be controllable. While studying several systems, including strong/strong, strong/weak, and weak/weak combinations of polyelectrolytes, it was observed that not all systems could be processed into saloplastic membranes. For the processable systems, expected trends were observed where a higher excess of one polyelectrolyte would lead to a more charged system, resulting in higher water uptake and better permselectivities. An anomaly was the polystyrenesulfonate–polyvinylamine system, which showed an opposite trend with a higher polycation ratio, leading to a more negative charge. Overall, we have found that it is possible to successfully fabricate saloplastic-based anion- and cation-exchange membranes with tunable charge densities through careful choice of polyelectrolyte combination and ratio of mixing.

## 1 Introduction

With the continuous progress in membrane science, research has been focused toward creating sustainable membrane materials and processes, and a host of new materials are available.^1,2^ While most alternatives are complex or non-viable, polyelectrolyte-based materials are very promising. Polyelectrolyte complexation (PEC) has become an increasingly important tool in membrane science in recent years, already leading to their commercial application as nanofiltration membranes.^3,4^

Dilute aqueous solutions of oppositely charged polyelectrolytes are applied in an alternating fashion on flat-sheet or hollow-fiber supports to form polyelectrolyte multilayers (PEMs). They are used as the active separation layer of the membranes. Alternatively, concentrated polyelectrolyte solutions can be phase-separated using a pH or salt trigger. This gives rise to sustainable porous membranes for micro, ultra, and nanofiltration by a method called aqueous phase separation (APS) in the absence of toxic and polar aprotic solvents.^5,6^ Complex coacervates of polyelectrolytes have also been spin-coated into layers to form a variety of porous structures.^7^ Clearly, multiple approaches exist to utilize PEC as the basis of sustainable membrane formation. Moreover, polyelectrolyte-based membranes contain ionic crosslinks that can be broken under the right pH and salt conditions, in principle allowing them to be recycled.^8^

One very clear outcome of the approaches described above is the high versatility that comes from using PEC as the basis for membrane fabrication. Different combinations of polyelectrolytes, including polystyrene sulfonate (PSS)–polyallylamine hydrochloride (PAH), PSS–polydiallyldimethyl ammonium (PDADMA), and polyacrylic acid (PAA)–PAH, have been studied well for various types of separations, leading to membranes with very different and specific properties.^9−11^ Moreover, studies have demonstrated an enormous control over membrane charge densities and through that their ion separation properties by tuning the pH salinity and fabrication approach.

One thing that was missing until recently was an approach to producing thick and fully dense polyelectrolyte films. Fully dense polymeric materials are typically fabricated using pressure and/or elevated temperature,^12^ for example, through compression molding methods such as extrusion. The Schlenoff group has demonstrated the processing of PEC by extrusion using saltwater. They were appropriately termed “saloplastics”, as salt dopes the complex while water plasticizes it, and the provision of heat in the extruder enhances the mobility of the polymer chains. However, these saloplastics are not devoid of pores.^13^ In our previous work, a hot-pressing method was devised to overcome this by applying pressures up to 200 bar.^8,14^ A well-studied system of two strong polyelectrolytes—PSS and PDADMA––was employed to facilitate this, and the plastic contained an excess of positive charge. The obtained plastic sheets were further used as anion and cation exchange membranes for ion selectivity.^14,15^ PSS–PDADMA anion exchange membranes displayed monovalent–divalent selectivities of 6.3 for sulfate over chloride ions, while PSS–PVH membranes showed monovalent–monovalent selectivities of up to 1.91 for potassium over sodium ions.

In the case of PSS–PDADMA, increasing the molecular weights of the individual polyelectrolytes was shown to increase the net charge of the complex formed between them.^8^ Higher molecular weights indicate longer chains, leading to more entanglements, and thus for the functional groups of the polyelectrolytes, it becomes more difficult to interact with an oppositely charged group. When both polyelectrolytes have high molecular weights, the net charge is the highest, leading to the best separation properties.^8^ A clear indication that, also for these newer polyelectrolyte-based membranes, there are tuning parameters to control their properties.

In the literature, charge variation of saloplastics has also been demonstrated by changing the salt concentration in the initial polyelectrolyte solutions.^16^ Shamoun et al. were able to achieve increased charge up to 24% by precipitating PSS–PDADMA complexes in 2.5 M NaCl solutions.^17^ This is an additional testimony that varying the quantities of parameters does influence the final charge of precipitates and, in turn, that of the saloplastics. However, for the application as ion exchange membranes (IEM), a dense structure is desired. The presence of a large number of counterions in the precipitate as well as the quantities of salt used led to crystallization and finally to pores and white membranes during initial trials. Hence, it does not result in IEMs, and this approach was not employed.

The use of saloplastics as IEMs requires significant charge densities, and being able to tune this property can also allow important control over physical and mechanical properties, and most importantly their separation properties. In this work, we study in detail the effects of two tuning parameters: the choice of the polyelectrolyte couple and the monomer ratio in which the polyelectrolytes are mixed. Initially, we study the processibility of eight different combinations of strong/strong, strong/weak, and weak/weak polyelectrolytes. For the polyelectrolyte couples that could be successfully processed into saloplastics, we varied the ratios of the repeat units while carefully studying their material properties and separation performance. We further compare the membrane properties to their commercially available IEM counterparts. Overall, we clearly demonstrate that both cation and anion exchange membranes can be made and that their charge densities can be effectively controlled by tuning the ratio of mixing and the choice of polyelectrolytes.

## 2 Materials and Methods

### 2.1 Materials

Poly(sodium 4-styrene sulfonate) (Na-PSS, Mw = 1000 kg mol^–1^, 25 wt % in H_2_O), chloride salt of poly(diallyldimethylammonium) (PDADMAC, Mw = 400–500 kg mol^–1^, 25 wt % in H_2_O), PAA, Mw = 250 kg mol^–1^, 35 wt % in H_2_O, NaCl (>99%), KCl (>99%), and KBr (>99%) purchased from Merck Nederland. Polyvinylamine (PVA-HCl) was purchased as Xelorex RS1300 (MW = 350 kg mol^–1^) from BASF Belgium and used as received. PAH-Cl Mw = 150 kg mol^–1^, 40 wt % in H_2_O was from Nittobo Medical Co., Japan. Hydrochloric acid, HCl (37%), and sodium hydroxide (NaOH pellets) were both purchased from Sigma-Aldrich. Milli-Q water from a Millipore Synergy Water Purification System was used to make polyelectrolyte solutions.

### 2.2 Preparation of Polyelectrolyte Complexes

Each combination of anionic and cationic polyelectrolytes was prepared differently in accordance with the ratio in which they are known to combine from literature as well as other specific observations. The total mass of the dry polymer was kept constant at 30 g L^–1^ for all the ratios in each combination.

#### 2.2.1 PSS–PDADMA Complex

Complexes were prepared in five different ratios of the repeat units—3:1, 2:1, 1:1, 1:2, and 1:3. In each case, two separate single-polyelectrolyte solutions were prepared for each ratio with 125 mM of KBr.

To prepare each polyelectrolyte solution, water was taken in a beaker to about half of the intended final volume of the solution. Salt was weighed and added to the water, and dissolved. Next, the calculated weight of the polyelectrolyte was added, and then water was filled to the intended volume. A stirrer bar was inserted and mixed well for 10 min to form a homogeneous solution. Finally, both solutions were combined by pouring them simultaneously into a third beaker under stirring and allowed to stir for 3 h. Afterward, each solution was allowed to rest for 24 h.

#### 2.2.2 PSS–PAH Complex

Individual solutions of Na-PSS and PAH were prepared in the ratios according to their repeat units, respectively,^6^ along with 100 mM KBr. The procedure is similar to Section 2.2.1.

#### 2.2.3 PSS–PVH Complex

Solutions of Na-PSS and PVH were individually prepared in ratios similar to the method described in Section 2.2.1, with a total of six ratios: 3:1, 2:1, 1:1, 1:2, 1:2.5, and 1:3.

#### 2.2.4 PAA–PDADMA Complex

Individual solutions of PAA and PDADMAC^18^ were prepared in the ratio 1:1 with respect to the repeat unit, similar to the method described in Section 2.2.1, in 50 mM NaCl instead of KBr.

#### 2.2.5 PSS–PEI Complex

Individual solutions of PSS and PEI were prepared in the ratio 2:1, similar to the method described in Section 2.2.2 with 100 mM KBr.

### 2.3 Hot-Press Molds

A Delrin (DuPont) sheet was cut into two rectangular plates of dimensions 150 × 100 × 6 mm^3^. On the bottom plate, a spacer was glued. It was cut out of a PTFE coated fiberglass sheet (Lubriglas-CHAP-1540) of thickness 0.122 mm with adhesive on one side (Figure S1), purchased from Reichelt Chemietechnik GmbH+ Co (Heidelberg, Germany). Narrow outlets were made on each edge to facilitate excess water, PEC, and air to escape. The top plate was used as it is. Together, they constitute a mold.

### 2.4 Centrifugation

The dispersed complex, with/without supernatant, was poured into centrifuge tubes and centrifuged using a Corning LSE compact centrifuge at 6000 rpm for 30 min. The supernatant was discarded. This was repeated at least twice (without adding new liquid in subsequent centrifugations) until the precipitate was compact enough to be handled and processed.

### 2.5 Hot-Pressing

An FV20R Rollie Driptech Rosin Press (purchased from FVR, Canada) was used to hot-press PEC. For this, 2–5 g of wet PEC was placed on the lower plate of the mold and closed with the upper plate. This mold was placed in between the aluminum slabs of the hot-press. The slabs of the hot-press were slowly closed together in such a way that they touched but were not subjected to any pressure. The heating was switched on, and the desired temperature for each PEC was set, leading to an increase from room temperature in 10–15 min. The mold was allowed to sit at this temperature for 10 min before a PEC-specific pressure was applied. The PEC was allowed to remain so for 5 min. Finally, the temperature was set to 25 °C to allow gradual cooling. When 25 °C was reached in ∼30 min, the pressure was released, mold opened, and the plastic PEC sheet was removed.

### 2.6 Water Content and Water Uptake

A film was weighed in storage condition, fully hydrated as well as dehydrated states, and the differences were compared with the latter to understand water uptake. For hot-pressed films of each combination of polyelectrolytes, films were cut into rectangular strips of 2 cm × 1 cm, and masses were recorded (*m*_sto_) at 37–40% humidity. These samples were then immersed in MilliQ water for 24 h, after which the masses were again recorded (*m*_wet_). Next, they were dried in a vacuum oven at 30 °C until a constant weight was recorded after about 24 h. The masses were recorded once again (*m*_dry_).

Similarly, the difference in storage (air) and dry masses of a membrane, taken as a ratio to the dry mass, gives the value of water content in ambient storage conditions, as shown by the following equation.
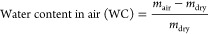


The difference in wet (water) and dry masses of a membrane, taken as a ratio to the dry mass, gives the value of water uptake. It is the water content in the film after equilibration in water, which is shown by the following equation.
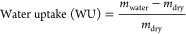


### 2.7 Ion Exchange Capacity

Potentiometric titrations were performed to determine the anion and cation exchange capacities, AEC and CEC, of each film, respectively.^19^ Then, depending on the type of membrane, the lower value was subtracted from the higher value to obtain the net ion exchange capacity (IEC).

To determine the AEC, the samples were first brought to the Cl^–^ form by soaking 0.2 g of a dry membrane in 150 mL of 1.0 M NaCl for 24 h. Next, the membrane was rinsed and soaked in MilliQ water for 90 min, during which the water was replaced several times to remove the sorbed NaCl. The Cl^–^ ions were replaced by SO_4_^2–^ ions by soaking the film in 50 mL of 1.0 M Na_2_SO_4_, during which the solution was replaced twice to ensure a complete exchange of Cl^–^ with SO_4_^2–^. These three solutions were combined, and the number of chloride ions released from the sample was determined by titration with 0.1 M AgNO_3_, whose endpoint was indicated by K_2_CrO_4_. The AEC was calculated as follows
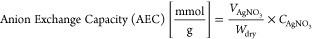


*V*_AgNO_3__ and *C*_AgNO_3__ are the volume and concentration of AgNO_3_, respectively.

To determine the CEC, the sample was brought to the H^+^ form by immersing 0.2 g of a dry membrane in 150 mL of 0.5 M HCl for 24 h. Next, it was rinsed in MilliQ water and soaked for 2 h, during which the water was replaced several times to remove sorbed HCl. Further, H^+^ ions were replaced by Na^+^ by soaking in 50 mL of 1 M NaCl, and the solution was replaced twice to ensure a complete exchange of H^+^ ions with Na^+^. These solutions were combined. The released quantity of H^+^ ions was determined by titration with 0.1 M NaOH in the presence of a pH electrode (Metrohm pH 491). The CEC was calculated by the following equation
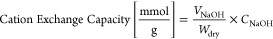


*V*_NaOH_ and *C*_NaOH_ are the volume and concentration of NaOH, respectively.

### 2.8 Permselectivity

The ability of a membrane material to allow the passage of counterions (anions for an anion exchange membrane and vice versa) while retaining co-ions is termed permselectivity. Permselectivity values quantitatively show the performance of a membrane in specific salt solutions. A test membrane was inserted between two chambers (Figure S2) with different concentrations of KCl solution in circulation. Each chamber had a calomel reference electrode (VWR, The Netherlands) measuring the voltage drop induced by the concentration gradient generated due to the difference in concentration on either side of the membrane. Numerically, the permselectivity is calculated as the ratio of the experimental voltage measured by electrodes to the theoretical Nernst potential for an ideally permselective membrane. The Nernst potential is given by
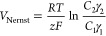
Here, *C*_1_ and *C*_2_ are the salt concentrations, while γ_1_ and γ_2_ are the activity coefficients. Using the above theoretically calculated voltage as well as the experimental voltage, the permselectivity is calculated as
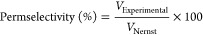


Each membrane was equilibrated in a 0.1 M KCl solution for at least 24 h before the permselectivity measurement.

### 2.9 Electrical Resistance

The electrical resistance offered by a membrane to the mobility of ions was measured using a six-compartment cell (Figure S3) made of plexi glass.^20^ A constant temperature of 25 ± 0.2 °C was maintained using a thermostatic bath. The two end compartments housed platinum-coated titanium electrodes to apply specific currents. Haber–Luggin capillaries connected to calomel reference electrodes were inserted into the central compartments to measure the potential drop across the test membrane. The test solution, for example, KCl, was circulated in the two central compartments using a peristaltic pump. A similar solution was circulated in each of the two adjacent compartments on either side. A 0.5 M solution of K_2_SO_4_ was continuously circulated in the end chambers. Commercial cation exchange membranes, Neosepta CMX from Astom Corporation (Japan), were placed in between every pair of chambers except the central ones, where the test membrane was inserted using a holder (Figure S4). The holder consists of two discs with gaskets to hold the membrane in place and avoid any leakage of the salt solution.

Each test membrane was equilibrated in the test solution for at least 24 h before testing. The electrodes supplied currents of 0–200 mA, resulting in voltage drops measured by the Haber–Luggin capillaries. An IV curve was plotted, and the slope was determined for the DC resistance. The solution resistance (obtained by measuring the resistance of the holder without a membrane) was subtracted from each value and multiplied by 0.785 cm^2^, the effective area of the membrane, to obtain the value of area resistance reported in this paper.

A 5 mA fixed amplitude AC signal was supplied, and the frequency was varied from 1 to 100 MHz while the response was recorded with a PGSTAT302N, Metrohm Autolab (The Netherlands) potentiostat. The impedance was measured with a minimum phase shift value in the frequency range of 100–1000 Hz to obtain the AC resistance. The AC resistance was subtracted from the DC resistance to obtain the resistance of the diffusion boundary layer.

### 2.10 Ion Selectivity

The ion selectivity of the IEMs is given by the following equation
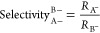
where  is that measured in ACl_*x*_ and  is the resistance measured in BCl_*y*_ solutions, and *x* and *y* are the valencies of A and B, respectively. Three monovalent ions (Li^+^, Na^+^, and K^+^) and two divalent ions (Ca^2+^ and Mg^2+^) were used as chloride salts in aqueous solutions to determine the resistances. All the solutions were made such that they had the same ionic strengths for a fair comparison.

### 2.11 pH Stability

Permselectivities of hot-pressed plastics were measured, and then the samples were stored in 1 M (pH 0) HCl (37%) or 1 M (pH 14) NaOH. After the duration of storage, the samples were rinsed thoroughly in MilliQ^21−23^ water and equilibrated in a solution identical to the permselectivity solution. The permselectivities were measured again. For samples that degraded, the experiment was repeated with the next number, for instance, pH 2 or 13, and so on until the stability range was determined for each sample.

### 2.12 UV–vis Spectroscopy

Opacity was assessed using a UV 1800 spectrophotometer from Shimadzu Corporation, Tokyo, Japan. Plastics were cut into strips of 8 × 32 mm^2^ and placed in the cuvette to measure the absorbance at 600 nm wavelength. The absorbance was recorded, and the opacity was calculated as follows, wherein *d* is the thickness of the sample
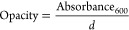


### 2.13 Thickness Measurements

All mentioned thicknesses were measured using a handheld series 293 micrometer from Mitutoyo Instruments. Each reported value is an average of at least five different measurements taken at random positions.

### 2.14 Commercial Membranes

The properties of hot-pressed plastic membranes were compared with those of commercial membranes, Neosepta AMX, CMX, and Neosepta ACS (obtained from Astom Corporation, Japan).

## 3 Results and Discussion

The results and discussion are divided into four parts, beginning with (a) the processibility of PEC into saloplastics, followed by (b) their physical and mechanical properties, (c) the effect of the ratio of mixing on the charge density and ion-exchange performance of the saloplastic membranes, and finally (d) the hot-pressed saloplastics are compared to the characteristics of common commercial membranes and discussed.

### 3.1 Processing Polyelectrolyte Complexes to Saloplastics

The processability of a complex depends mainly on the affinity between the polyelectrolytes, the compactness of the complex, and the molecular weights of the polyelectrolytes.^18^ While saloplastics have been made using combinations like PAA–PAH,^24^ most work has been done with PSS–PDADMA,^25−27^ including for their use as tissue scaffolds^26^ and IEM.^15^ The following combinations (Table 1) were chosen for this study comparing various polyelectrolyte couples, including strong/strong, weak/strong, and weak/weak couples as indicated. Their processability into a dense saloplastic was studied, while for a more successful system, also a range of monomer ratios was investigated as indicated.

**Table 1:** Summary of Tried Polyelectrolyte Combinations and Their Processibility

system	combinations	processible	comments	studied further?
strong–strong				
1	PSS–PDADMA			
	3:1	after centrifugation		yes
	2:1	after centrifugation		yes
	1:1	easiest processibility	stiff mozzarella-like	yes
	1:2	after centrifugation		yes
	1:3	after centrifugation		yes
strong–weak				
2	PSS–PVH		only dispersed aggregates	
	3:1	after centrifugation		yes
	2:1	after centrifugation		yes
	1:1	after centrifugation		yes
	1:2	after centrifugation		yes
	1:2.5	after centrifugation	clear supernatant	yes
	1:3	after centrifugation		yes
3	PSS–PAH			
	3:1	after centrifugation	sticky/pasty	yes
	2:1	after centrifugation	pasty	yes
	1:1	after centrifugation	pasty/clear supernatant	yes
	1:2	after centrifugation	pasty	yes
	1:3	after centrifugation	pasty	yes
4	PAA–PDADMA			
	1:1	yes, difficult	sticky, not reproducible	yes
5	PSS–PEI			
	1:1	no	burns at the temperature required to plasticize	no
weak–weak				
6	PAA–PAH			
	several ratios	no	gel-like complex	no
7	PAA–PVH			
	several ratios	no	gel-like complex	no
8	PEI–PAA			
	several ratios	no	fluid complex	no

#### 3.1.1 NaPSS–PDADMAC

The complexation and processing of the two strong polyelectrolytes, PSS with PDADMA, have been studied in the literature in several ways and for different applications. Their PEMs have been examined for properties such as hydrophilicity,^28^ ion transport,^29^ and doping, and designed for applications including drug delivery,^30^ bio-nanoparticle incorporation,^31^ and membranes.^32^ Porous free-standing PSS–PDADMA membranes have been demonstrated by solution casting.^5^ Shamoun et al. extruded stoichiometric complexes^27^ and evaluated the effects of factors such as salt concentration, temperature, kinetics, and diffusion.^16^ Thermal transitions in dried polyelectrolytes have also been studied.^33^ The current section of this paper focuses on varying the ratio of PSS and PDADMA repeat units and the resulting processability.

Five monomer ratios of PSS/PDADMA, 3:1, 2:1, 1:1, 1:2, and 1:3, were chosen, and complexes were made with KBr (125 mM) as a background salt (Figure S5). For the stoichiometric (1:1) ratio, the macrophase was easy to process, as in literature.^27^ The processing and properties of this complex have been studied in detail in our previous work.^15^ The plastics formed with ratios 2:1 and 1:2 were fragile compared to the stoichiometric complex, those formed at ratio 1:3 were even weaker, while the 3:1 plastic was extremely weak and the films folded and curled while handling. Hence, only small pieces of the 3:1 plastic were available for characterization. The hot-pressing conditions are shown in Table S1.

#### 3.1.2 NaPSS–PVH(Cl)

Na-PSS (strong polyanion) and PVH (weak polycation) were combined in monomer ratios of 3:1, 2:1, 1:1, 1:2, and 1:3. Unlike the PSS/PDADMA complex, this combination initially gave a milky dispersion of tiny complex particles in all the above ratios. Clear macro- and micro-phases were observed only for the ratio 1:2, which is close to complete phase separation. For the other ratios, a fraction of the particles settled when allowed to sit still for more than 24 h, while others remained suspended in solution. These complex particles are likely more charged and so small that sedimentation does not occur as expected. Also, 1:3 was visually less opaque than 1:1 and had a good precipitate. Hence, a 1:2.5 ratio was added to the sequence (Figure S6).

The ratios 3:1, 2:1, 1:1, and 1:3 were transferred into centrifuge tubes and centrifuged at 6000 rpm for 6 h. Better phase separation was seen, but the supernatants were still translucent, indicating the presence of PECs. Cycles of centrifugation and supernatant discharge were repeated a few more times, for 1 h each, until there was no more retrievable supernatant. Further, precipitates were not easily processible and were pasty. Videos S1 and S2 of the Supporting Information show the differences in the precipitates of PSS–PDADMA and PSS–PVH. The precipitates containing PDADMA are sturdy and difficult to tear, like a ball of mozzarella,^15^ but the PAH-containing PECs were softer and contained smaller aggregates, like concentrated cheese spread.

About 2.6 g of 1:2 PSS/PVH precipitate gave a hot-pressed 100 μm film with an area of 1.4 cm^2^. Reproducible saloplastics were obtained when the precipitate was placed in the Delrin mold at room temperature and gradually heated to 95 °C. Plasticization at 95 °C required about 15 min, after which it was pressurized to 200 bars and allowed to cool to room temperature (Table S1).

#### 3.1.3 NaPSS–PAH Complex

Multilayer studies have shown good selectivities and tunable properties for this combination of polyelectrolytes.^34^ Na-PSS (strong polyanion) and PAH (weak polycation) were combined in monomer ratios of 3:1, 2:1, 1:1, 1:2, and 1:3 (Figure S7). This led to a milky white phase in each ratio, unlike the more colloidal suspensions in the case of PSS–PVH mixtures. Some phase separation (although not compact) and a clear supernatant were observed only for the 1:1 ratio. Such a state does not allow processing, and hence all the ratios were centrifuged at 6000 rpm for 6 h. After discharging the supernatant, the centrifugation and supernatant discharge cycles of 1 h each were repeated until the complex was compact enough to be processed. In this way, for the ratio 1:1, a complex that was convenient to handle was obtained after at least four centrifugation steps. The other ratios needed longer centrifugation times due to the pasty nature of their precipitates and were even sticky in the case of 3:1.

All ratios yielded dense films. However, only the plastics obtained from the 1:1 ratio was sturdy with reproducible thicknesses. The ones from 1:3 and 3:1 had particularly huge variations in thickness at different points of the film (which is reflected in the tensile measurements in Figure S7).

A few other combinations were explored by replacing the strong polyanion NaPSS with a weak polyanion, PAA, paired with PDADMAC, PAH, PVH, and PEI to test strong–weak and weak–weak combinations. Also, PEI was combined with NaPSS. The obtained PECs were not easy to handle or process and their outcomes are explained in the Supporting Information and Figures S5–S11. Table 1 summarizes all of these combinations and ratios, and their ability to form complexes, showing their different levels of processibility. Hereafter, the three systems PSS–PDADMA, PSS–PVH, and PSS–PAH are studied in detail for their physical, mechanical, and ion-exchange properties.

### 3.2 Physical and Mechanical Characterization

We are interested in using saloplastics as IEMs and hypothesize that the material properties of the saloplastics made in the previous section affect the ion-exchange properties of these materials.^35^ Their pH stability relates to the possibility of their application in specific separations and processes.^36^ Furthermore, the percentage of water present in a plastic determines its storage and handling conditions. Very low values indicate brittleness and require extra care while handling them.^37^ The water uptake indicates swelling, and hence is instrumental in understanding the charge density and can be compared to permselectivities and IEC.^38^

**Figure 1:**
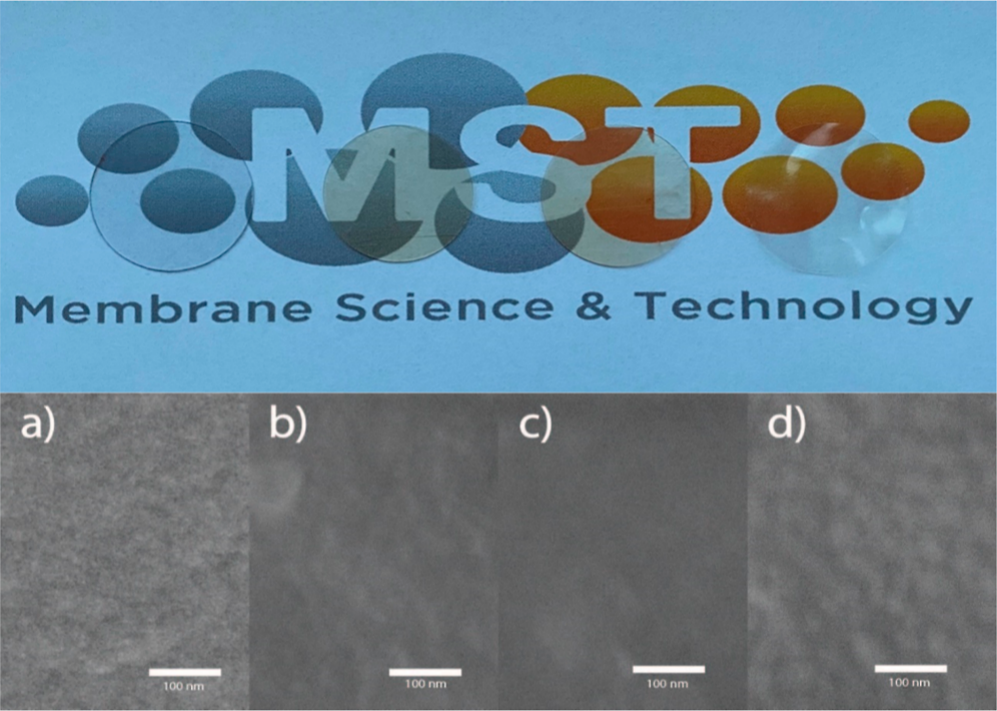
Photographs of the best ratio for each system, and FESEM images at 100,000× magnification of hot-pressed saloplastics from (a) 1:1 PSS/PDADMA, (b) 1:2 PSS/PVH, (c) 1:1 PSS/PAH, and (d) 1:1 PAA/PDADMA.

The hot-pressed plastics made from the best processible ratio in each of the three combinations (PSS–PDADMA, PSS–PAH, and PSS–PVH) along with PAA–PDADMA were photographed (Figure 1). They were all transparent and uniform and had only slight visual differences such as roughness and color. The stoichiometric PSS–PDADMA plastic was by far the most transparent and uniform, whereas the ones made from PSS–PVH and PSS–PAH had a yellow tint as the PAH and PVH solutions were yellow.^39^ PAA–PDADMA plastics were observed to have a matte appearance and to be more brittle. UV–vis measurements showed very low opacity values for all four films, confirming their transparency (Figure S16). Among these, the PSS–PDADMA films had the lowest opacities, while those of PSS–PAH and PSS–PVH were comparable.

Under the scanning electron microscope, they all had a dense structure with no observable pores on the nanometer scale, the limit of the field emission scanning electron microscopy (FESEM) (>1.2 nm). Further, no water permeability was recorded when such membranes were subjected to a dead-end permeability test, confirming that the membranes do not have pores.

**Figure 2:**
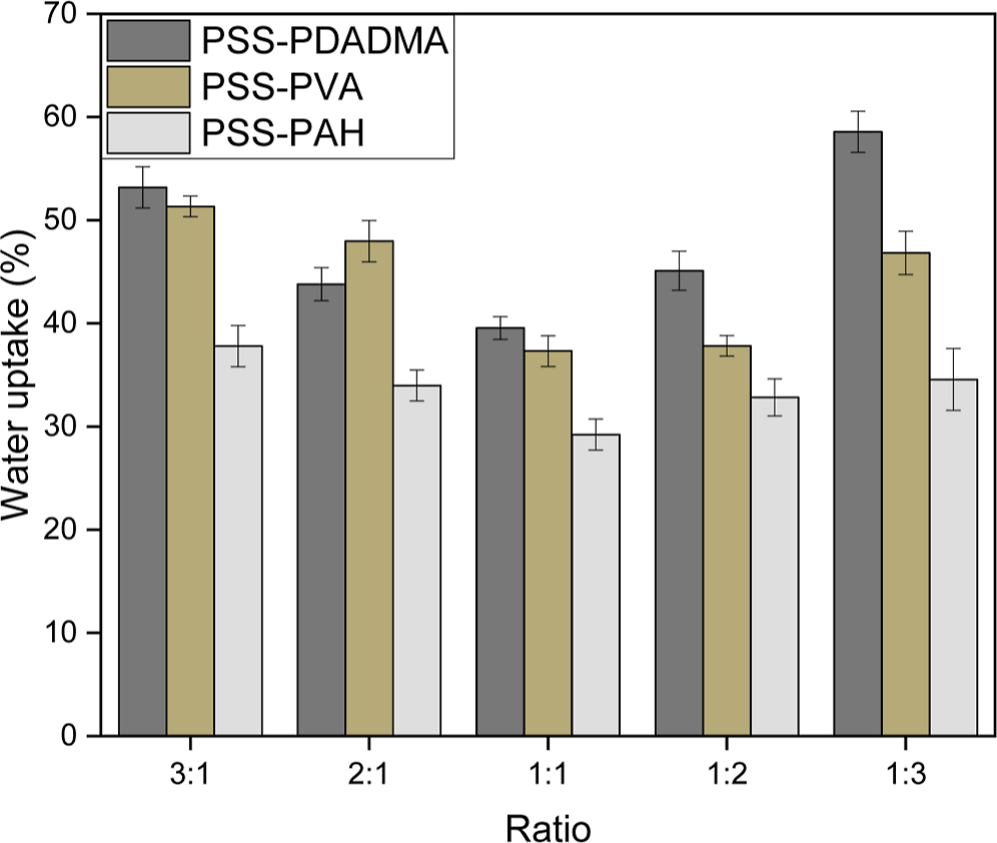
Water uptake of saloplastics made from different polyelectrolyte combinations and ratios. Error bars represent an average of at least three measurements.

Next, we determined the water uptake of the different saloplastics at varying ratios (Figure 2). It can be observed that as we deviate from stoichiometry, the uptake of water by the plastics increases without exception. Among the polyelectrolyte pairs, PSS–PAH was observed to take up the least water, while PSS–PDADMA took up the most.^40^ It has been observed for PEMs^41^ that the swelling of PSS–PAH is relatively constant with varying parameters such as salt concentration and ratio, whereas the percentage of water taken up significantly varies for PSS–PDADMA.^42^ The additional swelling for non-stoichiometric ratios is due to the association of some water molecules with extra charges due to the presence of one polyelectrolyte in excess.^43^ Their respective water contents are shown in Figure S17.

**Figure 3:**
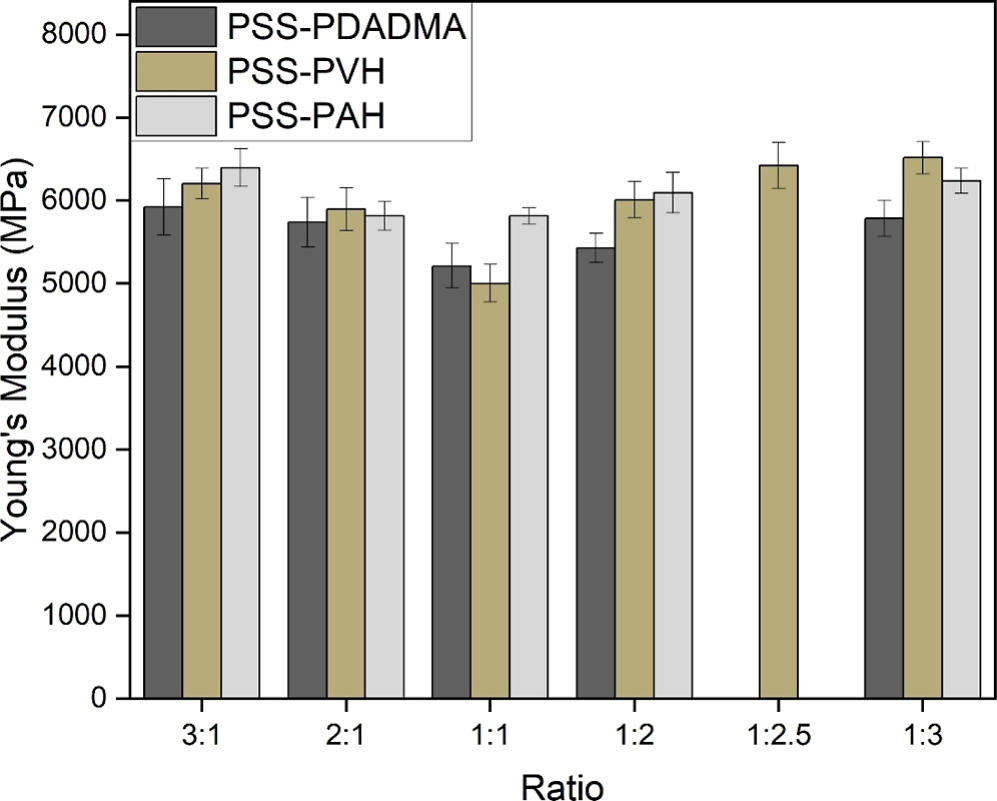
Tensile strengths of saloplastics in megapascals (MPa). Each value is an average of at least three measurements measured at a humidity of 42%. Refer to Table S2 for values and Figure S18 for stress–strain curves.

Tensile tests were performed to determine the strengths of the saloplastics and correlate this strength to their water contents. Across all three polyelectrolyte combinations, Young’s modulus is seen to be the lowest at, or near, stoichiometry (Figure 3). This is justified in the light of the water contents of the films, as a lower water content leads to a stronger and more brittle plastic, with a higher Young’s modulus.^44^ In the PSS–PDADMA series, plastics made with the 1:1 and 1:2 ratios show similar values. For PSS–PVH and PSS–PAH, their respective moduli for every ratio other than 1:1 are very similar with overlapping error bars. This is justified as they are nearly structural twins, with PAH having a single additional −CH_2_ group (Figure S19).^45^ PAA–PDADMA (1:1) had lower moduli of 3100 ± 380 MPa.

For several applications, IEMs need to be stable in a range of pH values. The stability of a stoichiometric PSS/PDADMA saloplastic in high and low pH was demonstrated in our previous work.^15^ A similar test with other PSS/PDADMA ratios showed that the 1:2 plastics were stable over a 14 day period in pH 1 and pH 14, while 1:3 and 2:1 showed signs of degradation at high pH (Table 2). The 3:1 plastics immediately began turning white, indicating pore formation. They disintegrated below pH 2 and above pH 9. All the ratios of the PSS–PVH, and PSS–PAH plastics were stable between pH 2 and 8, while the 2:1, 1:1, and 1:2 (also 1:2.5 for PSS–PVH) were also stable at pH 1 and up to 9. This is supported by the p*K*_a_ values of PAH and PVH around 9, beyond which they get deprotonated.^6^ Similar stabilities for PSS–PAH porous membranes have been reported by Baig et al.^46^

**Table 2:** pH Values Indicating the Stability Range of Hot-Pressed Saloplastics (Also Table S7)

system	ratio	3:1	2:1	1:1	1:2	1:3
1	PSS–PDADMA	2–9	1–12	1–14	1–14	1–12
2	PSS–PVH	1–8	1–8	1–9	1–9	2–9
3	PSS–PAH	2–8	1–8	1–9	1–9	2–8

### 3.3 Characterization as Ion-Exchange Membranes

The discussions in the previous section focused on properties pertaining to the physical and chemical stability of the materials, but the presence of charges introduces further complexity. High mechanical strengths reflect good crosslinking density but cause an increase in electrical resistance. Densely packed fixed charges reduce the electrical resistance but lead to a high degree of material swelling and therefore reduce mechanical stability. Therefore, the optimization of a material as an IEM involves a trade-off between physical properties and the density of fixed charged groups.^47^

IEMs depend on charged sites in the material to repel co-ions and to attract counterions to transport the latter across the membrane. Three characterization methods have been employed to link their charged nature to their separation properties. The amount of charged sites is represented by the IEC in mmol g^–1^ as measured by an acid–base titration method. The resistance to the passage of counterions is represented by the ohmic resistance (Ω cm^2^), and finally, the permselectivity gives a clear indication of the membrane selectivity.

**Figure 4:**
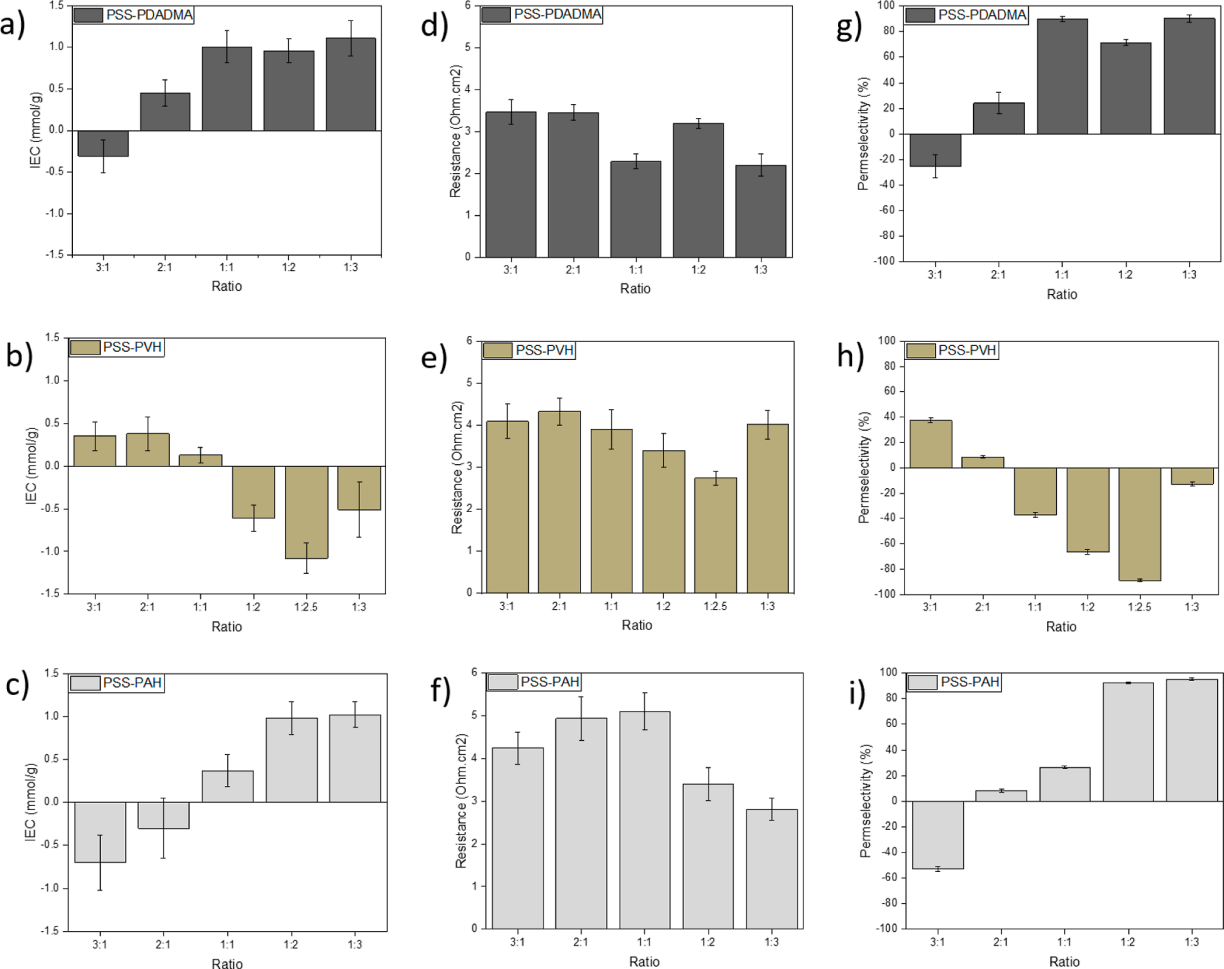
Comparison of the IEC (a–c), resistances (d–f), and permselectivity (g–i) of membranes made with different ratios of PSS–PDADMA, PSS–PVH, and PSS–PAH. Error bars represent an average of at least two values. For the IEC, negative values indicate a negatively charged membrane. Resistance offered to the passage of counterions by each membrane after subtracting the solution resistance normalized with area. All values are presented in Ω cm^2^. Each value is an average of at least three measurements. For the permselectivities, error bars represent an average of at least two values. Positive or negative values refer to positively or negatively charged membranes, respectively.

For the PSS–PDADMA system, the ratio 1:1 was measured to have a net IEC of 1.01 ± 0.2 mmol g^–1^.^15^ With an increase in PDADMA, the positive charge does not increase significantly as would be expected even when doubling or tripling the amounts. Table S3 indicates that altering the monomer ratio in the mixing solutions does not necessarily alter the ratio in which they complex. Doubling or tripling the amount of PSS (2:1 and 3:1) decreased the IEC significantly (Figure 4a–c). The negative sign here indicates that the net charge on the saloplastic is negative. That means that for these ratios, it was possible to control the resulting saloplastic charge by simply changing the monomeric ratio in the solution.

A similar study was conducted by Durmaz et al. for porous membranes of PSS–PDADMA with ratios closer to 1:1, namely 1:0.8, 1:0.9, 1:1, 1:1.1, and 1:1.2, through APS. Here, the zeta potential measurements revealed an excess of positive charge with ratios when PDADMA was in excess, as well as at the 1:1 ratio, while the ones with excess PSS gave negatively charged membranes.^5^ The saloplastic PSS/PDADMA system is similar, however, with the shift from positive to negative charge occurring between ratios 3:1 and 2:1. This further shows that varying the ratio of PSS/PDADMA clearly allows controlled charge variations.

The PSS–PAH system had a similar trend to PSS–PDADMA and gave the best precipitate at a ratio of 1:2. It is probably the closest to stoichiometry as the chains in the weak polyelectrolyte, PAH, are not completely charged. Also, some chains are discharged in the supernatant that could not be retrieved. The IEC value of 0.98 ± 0.2 mmol g^–1^ is reasonable, and an increase in the cationic polyelectrolyte PAH to a 1:3 ratio does not significantly change this number. On the other hand, the system nearly neutralizes at the 1:1 ratio, and further increases in PSS led to a net negative charge in an increasing trend. Compared to the PSS–PDADMA system, the ratios shift to the right, but the trend remains the same overall. For PSS–PAH, it does become much more clear that both positive and negative saloplastics can be prepared by simply varying the monomeric mixing ratio of the initial complexes.

Interestingly, the PSS–PVA system shows a contrasting trend to PSS–PAH and PSS–PDADMA. This was also the system wherein the supernatants were not totally transparent even after repeated centrifugation, indicating that some chains are lost when they are disposed of. A lot of positive chains likely leave as small complexes in the supernatant, such that the charge of PSS becomes dominant in the complex, leading to mostly negatively charged membranes.

The ratio that was easiest to process was 1:2.5 with an IEC of −1.1 ± 0.2 mmol g^–1^. A slight variation to 1:2 and 1:3 decreased the values, but the plastics remained negative. However, when the PSS^–^ was further increased, the saloplastics were near neutral or slightly negative. Vinyl cations are generally considered unstable, and although they only slightly vary from their allyl counterparts, their behavior can be different.^48^

The resistance offered to the flow of ions was tested by supplying current across a membrane and measuring the voltage in a 0.5 M KCl solution.^49^ The lowest resistances to the transport of counterions were observed (Figure 4d–f) for the 1:1 and 1:3 ratios in PSS–PDADMA, 1:2 and 1:2.5 for PSS–PVH, and 1:2 and 1:3 for PSS–PAH. In general, the values lie between 2.20 ± 0.30 and 3.41 ± 0.40 Ω cm^2^ (Table S4). These are comparable to common commercial cation exchange membranes and slightly lower as compared in Table 3.

**Table 3:** Summary of the Best Membranes in This Work^a^

summary	best ratio	thickness (μm)	Young’s modulus (MPa)	water uptake (%)	ion exchange capacity (mmol g^–1^)	charge density (mmol g^–1^)	area resistance (Ω cm^2^)	permselectivity (AEM/CEM) (0.03/0.15 M KCl) (%)
saloplastic membranes								
PSS–PDADMA	1:1	104 ± 4	5200 ± 280	40 ± 3	1.1 ± 0.3	0.79	2.29 ± 0.3	89 ± 2 (AEM)
PSS–PVH	1:2.5	105 ± 3	5600 ± 280	37 ± 2	1.1 ± 0.2	0.80	2.75 ± 0.2	88 ± 1 (CEM)
PSS–PAH	1:2	108 ± 6	6100 ± 240	32 ± 4	0.9 ± 0.2	0.68	3.40 ± 0.4	93 ± 1 (AEM)
commercial membranes								
Neosepta-AMX	NA	141 ± 6	3900 ± 190	19 ± 2	1.6 ± 0.2	1.34	2.40	94 (AEM)
Neosepta-ACS	NA	127 ± 3	3600 ± 220	31 ± 3	1.7 ± 0.3	1.30	3.80	95 (AEM)
Neosepta-CMX	NA	170 ± 9	4300 ± 160	24 ± 1	1.5 ± 0.3	1.21	3.00	97 (CEM)
Selemion-CMV	NA	120 ± 3	1900 ± 310	32 ± 4	1.6 ± 0.2	1.21	3.60	95 (CEM)

a Their main properties are compared to commonly used commercial IEMs. All the measurements, including those of the commercial membranes, were performed in our labs. Error bars indicate an average of at least two measurements, and more if a significant variation was observed. Refer to Figure S20 for structures of polyelectrolytes and Table S6 for PAA–PDADMA.

The selectivities to counterions were measured by placing membranes between two chambers with circulating KCl salt solutions, one with 0.03 M and another with 0.15 M, while recording the voltages. The calculated permselectivities are tabulated in Table S5 and plotted in Figure 4g–i.

We find a very strong correlation between the measured IEC values and the resulting permselectivity values, especially for the PSS–PDADMA series. The highest permselectivity of 90.4 ± 2.8% was observed for the ratio 1:3, comparable to 1:1. Nevertheless, the mass (assay) of the PEC precipitate obtained for 1:3 is much lower than in the stoichiometric case, which shows the loss of some of the excess PDADMA into the discarded supernatant. Further, the water uptake and tensile strengths also favor the 1:1 saloplastic, making it more viable. At 1:2, it drops by ∼20%.

For PSS–PVH, the ratios 1:2.5 and 1:2 are favorable with −89.1 ± 1.1 and −66.2 ± 2.0% respectively. It has been reported by Fu et al. that their PSS–PVH complex precipitates had an excess of negative charge in them.^18^ Nearly neutral plastics are obtained at a ratio of 2:1, while they are positive beyond this. Membranes made with the PSS–PAH system displayed reproducible permselectivities of 95.2 ± 0.9 and 92.6 ± 0.6% at 1:3 and 1:2 ratios, respectively. However, 1:2 is a better choice for usage due to the higher yield of PEC and ease of handling when wet.

Ideally, a solid lump-like precipitate is ideal to process. However, most ratios of all the combinations give small nanoscale aggregates with supernatants that are not clear. This shows their tendency to not mix in the ratio that we initially intend them to, leading to unexpected effects such as in PSS–PVH. Although predicting such behavior proved difficult, this is interesting and still shows the ability to change the charge density of these systems by tuning the two parameters—polyelectrolyte type and ratio.

### 3.4 Discussion and Comparison to Commercial Membranes

Based on the various ratios and combinations that were made and discussed above, the best ones were chosen based on a trade-off between easy processibility and good permselectivity.

In general, PSS–PDADMA plastics were the most sturdy with the 1:1 membrane being the best. The 1:3 membrane is equally good, but much material is wasted in the process of obtaining the saloplastic. PSS–PVH displays the best cation exchange properties at 1:2.5 with competitive resistance and mechanical properties. The PSS–PAH system displays low resistance as well as good permselectivity at 1:2, while higher amounts of PSS do not lead to an ion-selective saloplastic.

The targeted thickness in each of the saloplastic membranes was 100 μm. While the PSS–PDADMA and PSS–PVH systems were reproducibly very close to the target thickness, PSS–PAH had a slightly larger error.

Table 3 compares the properties of these ion-selective saloplastics with those of common commercial membranes. The current work showcases two anion- and cation-exchange membranes each, compared to the same number of commonly used commercial membranes. Such a comparison allows the understanding of the target values of each membrane property. Among the physical properties, the saloplastics are transparent and non-reinforced, as opposed to most commercial ones that make use of a mesh or substrate. Saloplastic membranes are sturdy with Young’s moduli generally higher than commercial membranes, although they could be reinforced for further stability.^8^ Another advantage is the ability to tune the thicknesses by simply changing the thickness of the mold, without any additional modification required.

The water uptake values of commercial ones are comparatively lower (19–32  %), which leads to better utilization of the charges by retaining a higher charge density.^50^ The saloplastic ones, on the other hand, take up more water (>32%), which may be improved by using crosslinkers such as glutaraldehyde.^51^ Further, the net IEC is about 1 mmol/g as against an upward of 1.5 mmol g^–1^ for commercial membranes.^52,53^ The combination of lower IEC and a higher degree of swelling also explains the somewhat lower permselectivities for the saloplastic membranes. This is reflected in the charge densities of PSS–PDADMA and PSS–PAH, which are 60% of their commercial counterparts, and that of PSS–PAH is 56%. Indeed, permselectivities are modest at up to 90% for PSS–PDADMA and PSS–PVH membranes, but better for the less swollen PSS–PAH with a permselectivity of 94%. The respective permselectivities for commercial membranes are still higher, ranging between 94 and 97%. The ion exchange performances of saloplastic membranes are in the range of commercial ones, and they show lower resistance to the transport of counterions, except in the case of PAA–PDADMA due to its inhomogeneity.

The four polyelectrolyte pairs, complexed and pressed to saloplastics, are testimony to the possibility of many more such combinations being processed into dense sheets. A careful study of each such combination may allow specific functionalities and properties for niche applications, such as the monovalent selectivity of Cl^–^/SO_4_^2–^ or K^+^/Na^+^.^14,15^ The demonstrated role of the polyelectrolyte pairs in membrane science, in general, has facilitated a lot of work in PEM-based hollow fiber membranes^10,54^ and more recently in APS of porous membranes.^55,56^ Further, expanding these to reverse osmosis and ion-exchange may require tolerance to higher pressures and/or extreme conditions such as pH or temperature.^57,58^ In addition, specific abilities to separate particular ions can be highly beneficial. Stoichiometric PSS–PDADMA saloplastics are pH stable and are demonstrable for monovalent–divalent anion selective properties favoring chloride over sulfate ions. Utilization of tailor-made polyelectrolytes with different functional groups can further broaden the scope of such separations. Furthermore, bipolar membranes may be fabricated by combining oppositely charged saloplastic membranes by the same hot-pressing approach with an additional step. These could be supportive in the transition from the use of harmful solvents toward green ways of membrane production.

Overall, varying the ratio of monomers in PEC allows much control over the charge in the saloplastic membranes. Incorporating different polyelectrolytes further allows tunability of their properties and can lead to a wide array of options, as observed in PEM membranes. The method is versatile and offers a range of sustainable possibilities for further research in dense membranes. Here, the focus has been on the use of dense saloplastics as IEMs, but we expect that these materials could be interesting for other applications.

## 4 Conclusions

Dense saloplastics made by hot-pressing PEC have demonstrated interesting ion-exchange properties. To understand their characteristics better and to tune their properties, varying the complexation parameters is vital. This work demonstrates the possibility to control the charge density of saloplastic-based IEMs by tuning the ratio of monomer units across different polyelectrolyte pairs. Other properties of saloplastics are also influenced, such as the water content, tensile strength, pH stability, and electrochemical properties. However, an increase in the monomer ratio of one of the polyelectrolytes does not always guarantee a change in the net charge. While with PSS–PDADMA and PSS–PAH, an increase in positive polyelectrolyte content led to positively charged saloplastics and vice-versa, the PSS–PVH combination showed an unexpected opposite trend. For the PSS–PDADMA, PSS–PAH, and PSS–PVH membranes, the 1:1, 1:2, and 1:2.5 monomer ratios were, respectively, the best combinations to form IEM. Their tensile strengths were typically higher than those of commercial membranes, while their resistances and permselectivities were in the same range. The higher swelling and lower IEC could be improved by crosslinking. They represent a new sustainable and highly tunable avenue to membrane formation and clearly display their possibility to compete with the performances of their commercial counterparts.

## Supporting Information Available

Resistance measurement setup, permselectivity setup, UV–vis chart, water uptake, electrochemical characterization tables, and photographs of hot-pressed polyelectrolyte complexes and plastics (PDF)
(PDF)
PSS–PDADMA complex (MOV)
PSS–PVA complex (MOV)

## Supplementary Material

la3c00497_si_002.pdf

la3c00497_si_003.mov

la3c00497_si_004.mov

## Abbreviations

Na-PSS: sodium salt of polystyrene sulfonic acid
PAH (PAA-HCl): poly(allylamine hydrochloride)
PVH (PVA-HCl): poly(vinylamine hydrochloride)
PDADMAC: poly(diallyl dimethyl ammonium chloride)
PAA: poly(acrylic acid)
PEI: poly(ethylene imine)
IEC: ion exchange capacity
AEC: anion exchange capacity
CEC: cation exchange capacity
PEC: polyelectrolyte complex
PECOV: polyelectrolyte coacervate
PEM: polyelectrolyte multilayers
