# Supplementary material for: Control over Charge Density by Tuning the Polyelectrolyte Type and Monomer Ratio in Saloplastic-Based Ion-Exchange Membranes
Source: Langmuir. 2023 May 1;39(19):6874–84. doi: 10.1021/acs.langmuir.3c00497 (PMC10193576; doi:10.1021/acs.langmuir.3c00497)
Supplement: Supplementary file 2 — la3c00497_si_002.pdf [file la3c00497_si_002.pdf]

Control Over Charge Density by Tuning the Polyelectrolyte Type and Monomer Ratio in Saloplastic Based Ion-exchange Membranes

Ameya Krishna B[a,b], Wiebe M. de Vos[a], Saskia Lindhoud[b,*]

[a]Membrane Surface Science, Membrane Science and Technology, MESA+ Institute of Nanotechnology, University of Twente, 7500 AE, Enschede, Overijssel, The Netherlands

[b]Department of Molecules and Materials, University of Twente, 7500 AE, Enschede, Overijssel, The Netherlands

SUPPLEMENTARY INFORMATION

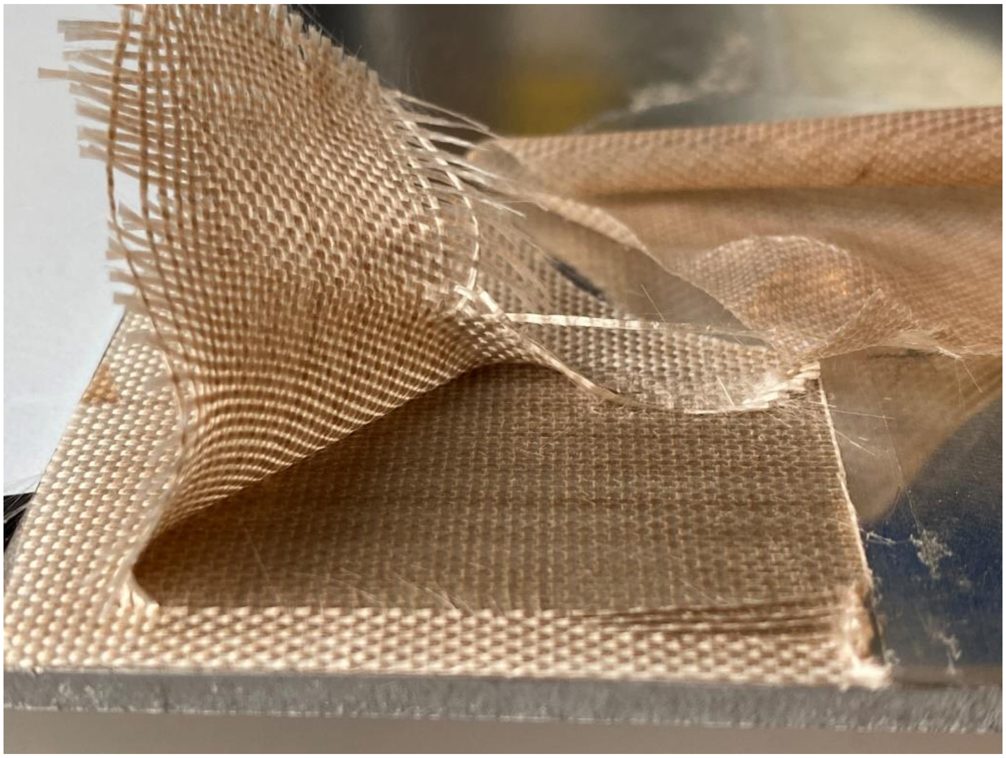

Figure S1. Glass fibre reinforced Teflon sheet as spacer glued to the edges of a Delrin plate to create moulds.

System	Combination	Max Temperature	Pressure
	Strong-Strong	°C	bar
**1**	PSS-PDADMA		
	3:1	92	50
	2:1	92	100
	1:1	92	300
	1:2	92	100
	1:3	92	100
	Strong-Weak		
**2**	PSS-PVH		
	3:1	90	100
	2:1	90	100
	1:1	90	100
	1:2	95	200
	1:2.5	95	200
	1:3	95	100
**3**	PSS-PAH		
	3:1	90	50
	2:1	90	100
	1:1	95	200
	1:2	95	200
	1:3	90	50
**4**	PAA-PDADMA		
	1:1	80	200
**5**	PSS-PEI		
	1:1	70-95	1-250
	Weak-Weak		
**6**	PAA-PAH<br>Several ratios	70-95	1-100
**7**	PAA- PVH<br>Several ratios	70-95	1-100
**8**	PEI-PAA<br>Several ratios	70-95	1-100

Table S1. Summary of hot-pressing conditions for all combinations presented in this work.

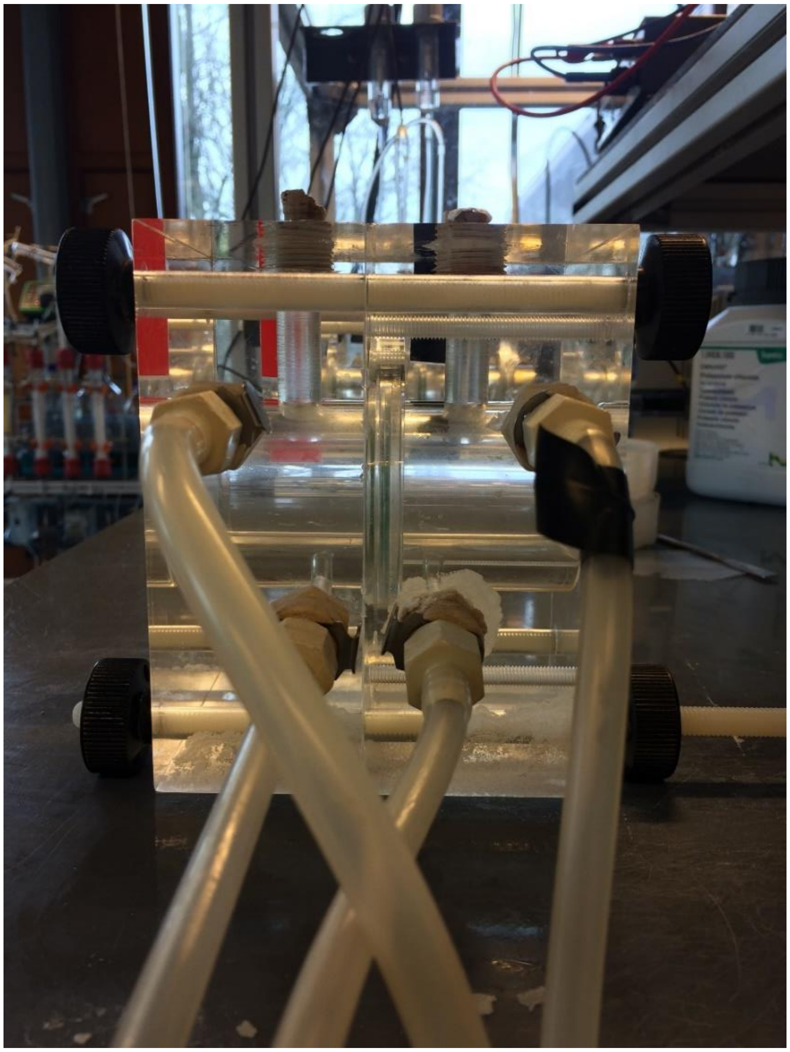

Figure S2. Permselectivity setup. (Line diagram of the setup available from our previous publication[1]).

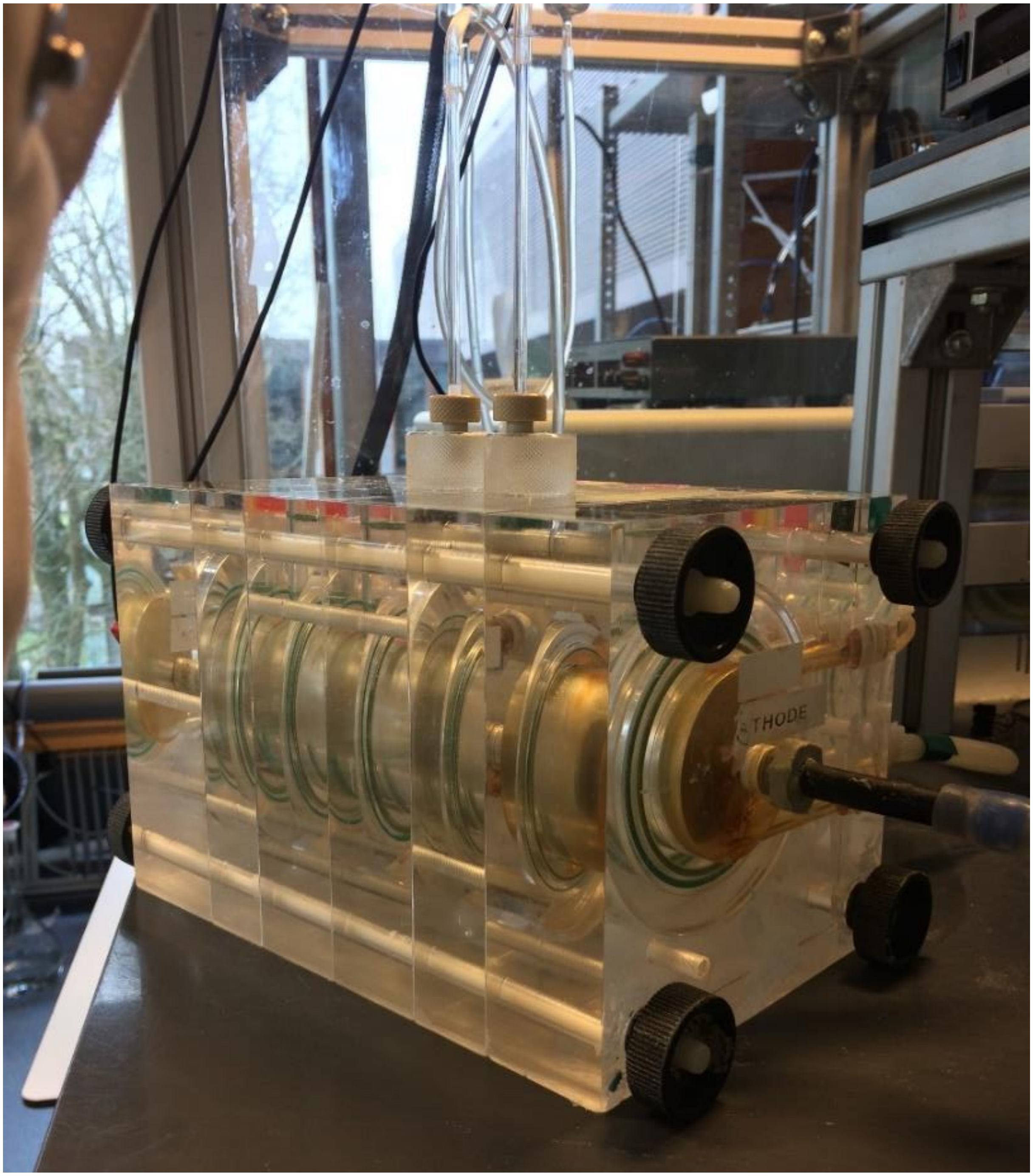

Figure S3. Resistance setup. Six chamber plexiglass cell with central chambers containing the circulating salt solution for testing. Electrodes supply current and voltage is measured by Haber Luggin capillaries. (Line diagram of the setup available from our previous publication[2]).

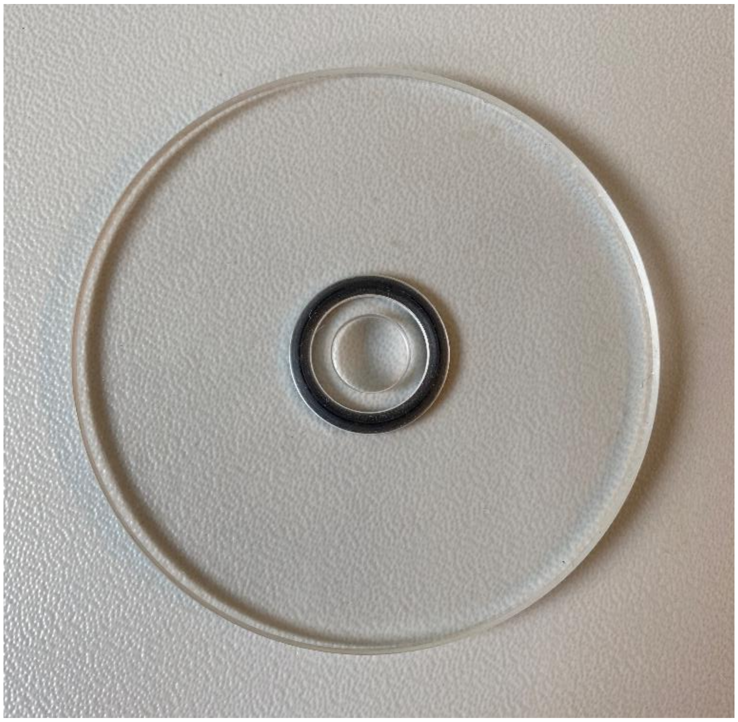

Figure S4. Holder for a test membrane to be inserted between chambers to measure permselectivity or resistance. The picture shows one of the two halves between which the membrane is placed and sealed by the black gaskets shown around the central opening.

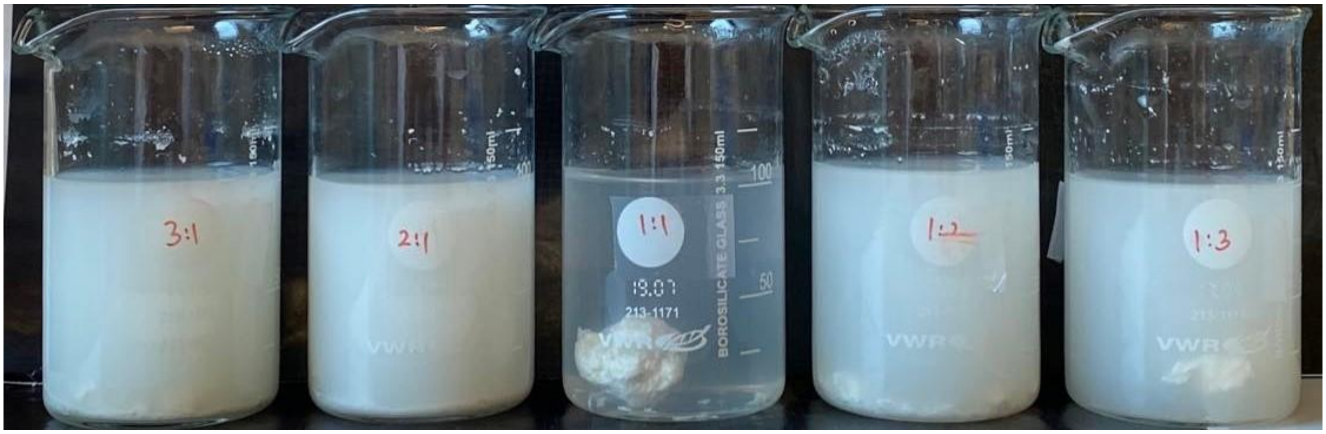

Figure S5. PSS: PDADMA complexes in monomer ratios 3:1, 2:1, 1:1, 1:2, and 1:3 in the presence of 125 mM KBr. Multiple batches were made to ensure reproducibility.

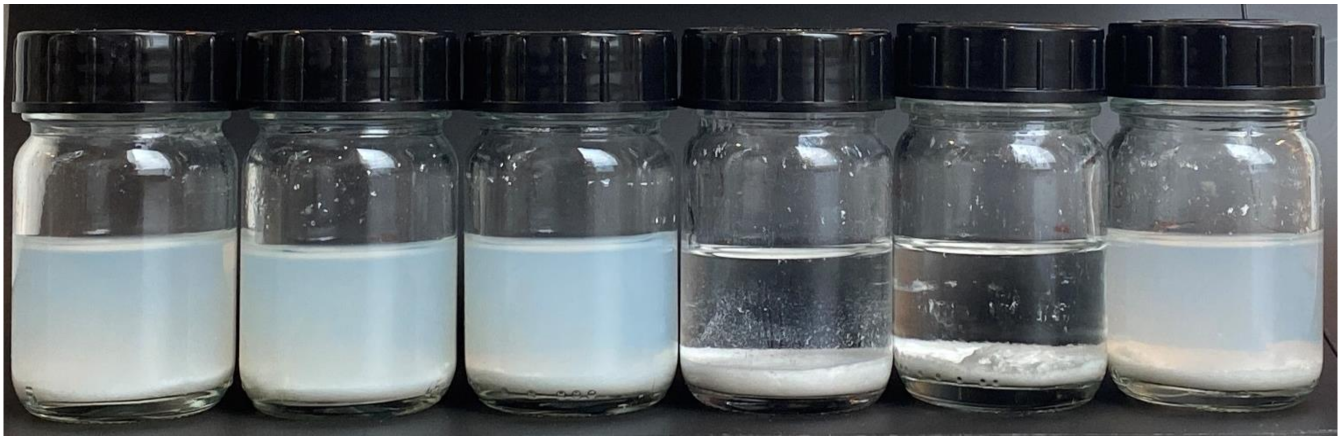

Figure S6. PSS-PVH complex in different ratios 3:1, 2:1, 1:1, 1:2, 1:2.5, and 1:3. Multiple batches were made to ensure reproducibility.

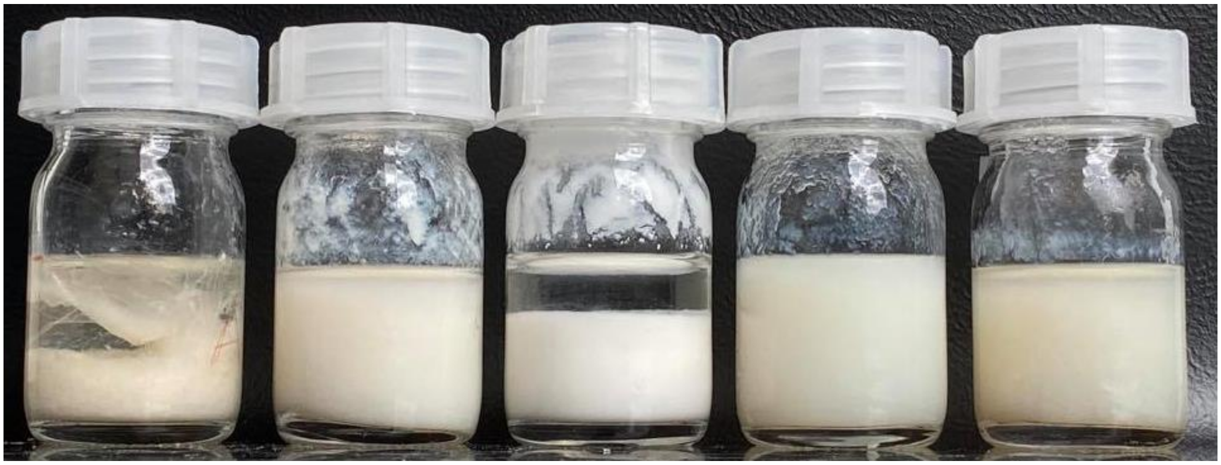

Figure S7. PSS-PAH complex in different monomer ratios, 3:1, 2:1, 1:1, 1:2, and 1:3. Multiple batches were made to ensure reproducibility

Qualitative aspects of PSS-PDADMA series:

For the stoichiometric (1:1) ratio, the macrophase consisted of a precipitate that was easy to handle and process, similar to observations in literature.[3] The processing and properties of this complex have been studied in detail in our previous work.[1]

The other 4 ratios also led to a macrophase separation, but milky supernatants indicated the presence of smaller PECs containing one of the polyelectrolytes in excess. These systems required centrifugation before hot-pressing, after which the supernatants were translucent.

The plastics formed with ratios 2:1 and 1:2 were fragile compared to the stoichiometric complex, those formed at ratio 1:3 were even weaker, while the 3:1 plastic was extremely weak and the films folded and curled while handling. Hence, only small pieces of the 3:1 plastic were available for characterization. The hot-pressing conditions are shown in **Table S1**.

Nature of the complexes formed:

PAA-PDADMA complex. It is known from literature that the interaction between these two polymers is not very strong.[4,5] Several ratios were attempted but were difficult to bring to a processible state. The complex formed with a 1:1 ratio was a lump of sticky substance glued to the stirrer bar and the beaker. The supernatant was observed to be whitish-grey and cloudy. Washing and removing the excess gluey substance made it reasonably processible. It was then hot-pressed at a relatively low temperature of 75°C. The plastic thus formed was sturdy in the dry state, but was weak in the wet state in comparison to the other polyelectrolyte pairs. When dry, it was observed to crack easily when bent. Also, in higher salt concentrations (>1M NaCl), the film was unstable (**Figures S8-S10**).

PSS-PEI complex.

PSS:PEI was combined in a 1:2 monomer ratio. The precipitate was very brittle and it was difficult to phase separate from the supernatant. After several rounds of centrifugation, a brittle lump was obtained. Hot-pressing failed multiple times with Delrin, and charring of the film was observed at temperatures above 85°C. Reduction of the temperature below 70°C flattened the precipitate but did not lead to plasticization. Between 70°C and 85°C, brittle, inhomogeneous, partially plasticized material was

obtained, which was difficult to handle or use (**Figures S11-S12**). Hence, processing of the PSS:PEI complex into a saloplastic was unsuccessful.

Weak-weak combinations. Apart from the above mentioned ones, other combinations consisting both weak polyelectrolytes, such as PAA-PAH, PAA- PVH, and PEI-PAA were experimented with. They did not yield suitable complexes even after multiple trials (**Figure S15**) and hence were not considered for processing. PAA-PAH was processible but the saloplastic was unstable already at atmospheric humidity (**Figure S13-S14**).

PAA-PDADMA saloplastic behaviour:

In the case of PAA-PDADMA, the saloplastics were not always homogenous with thicknesses varying up to ±20% in some cases. Further, their fragile nature required careful mounting on the holder (**Figure S9**) before equilibration as it was not stable and would curl or fold otherwise. The PAA-PDADMA plastics had the largest deviation in average thickness due to the nature of the polyelectrolyte complex. The membranes had a negative charge, with ion exchange capacities of -0.68 ± 0.36 mm g^{-1}. The fragile nature and instability in 1M NaCl did not allow the accurate measurement of ion exchange capacity for this combination of polyelectrolytes. Hence the IEC number is only a ballpark estimate. The resistance to was measured to be 4.7 ± 0.36 $\Omega \cdot cm^2$, while the permselectivity was -58 ± 5.1 %.

Overall, the PAA-PDADMA system may need other solvents such as ethanol in the polyelectrolyte solutions in order to form processible complexes.[6]

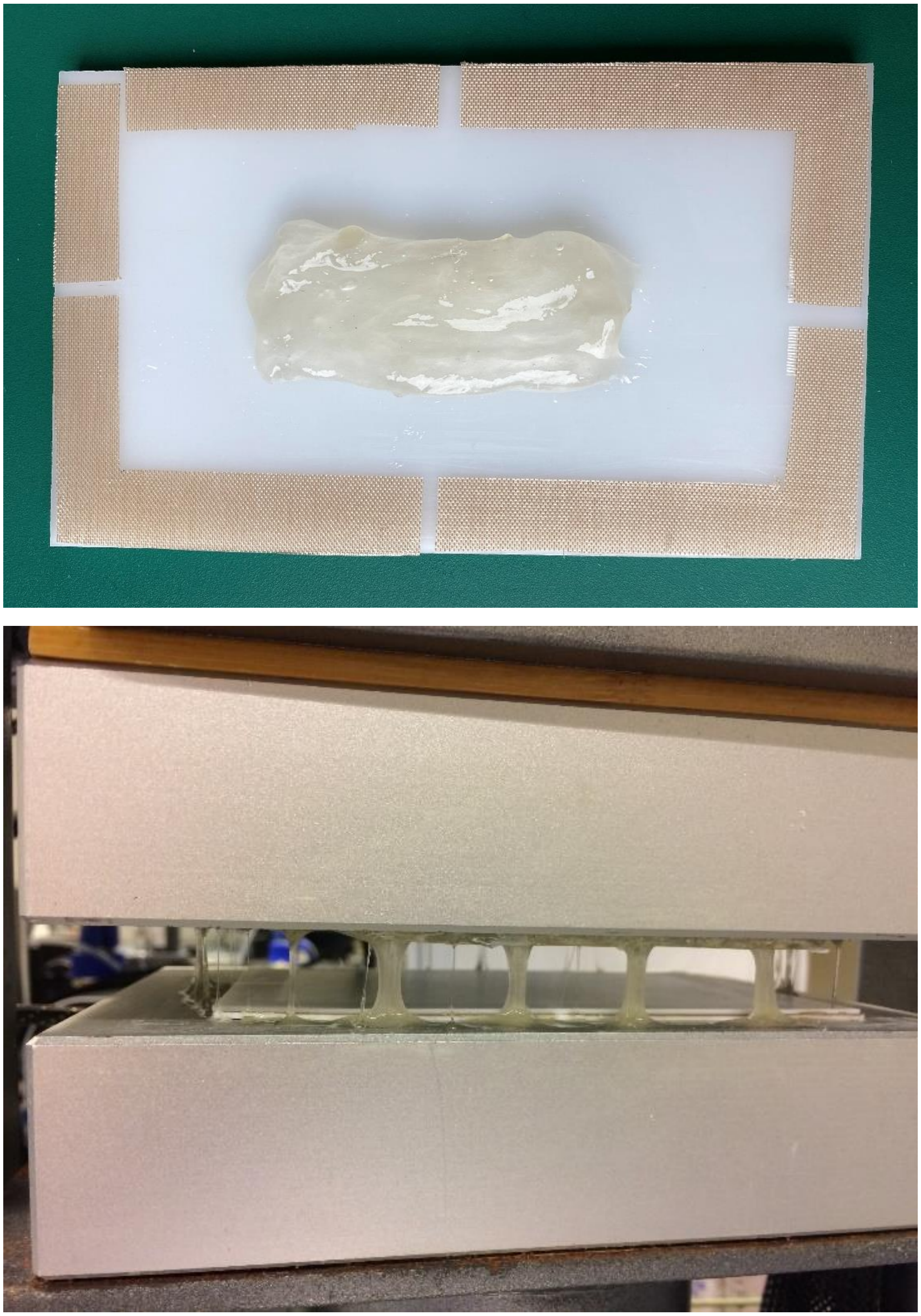

Figure S8. Photograph of initial hot-pressing of PAA-PDADMA before removing outer gel-like layer, making it nearly impossible to process.

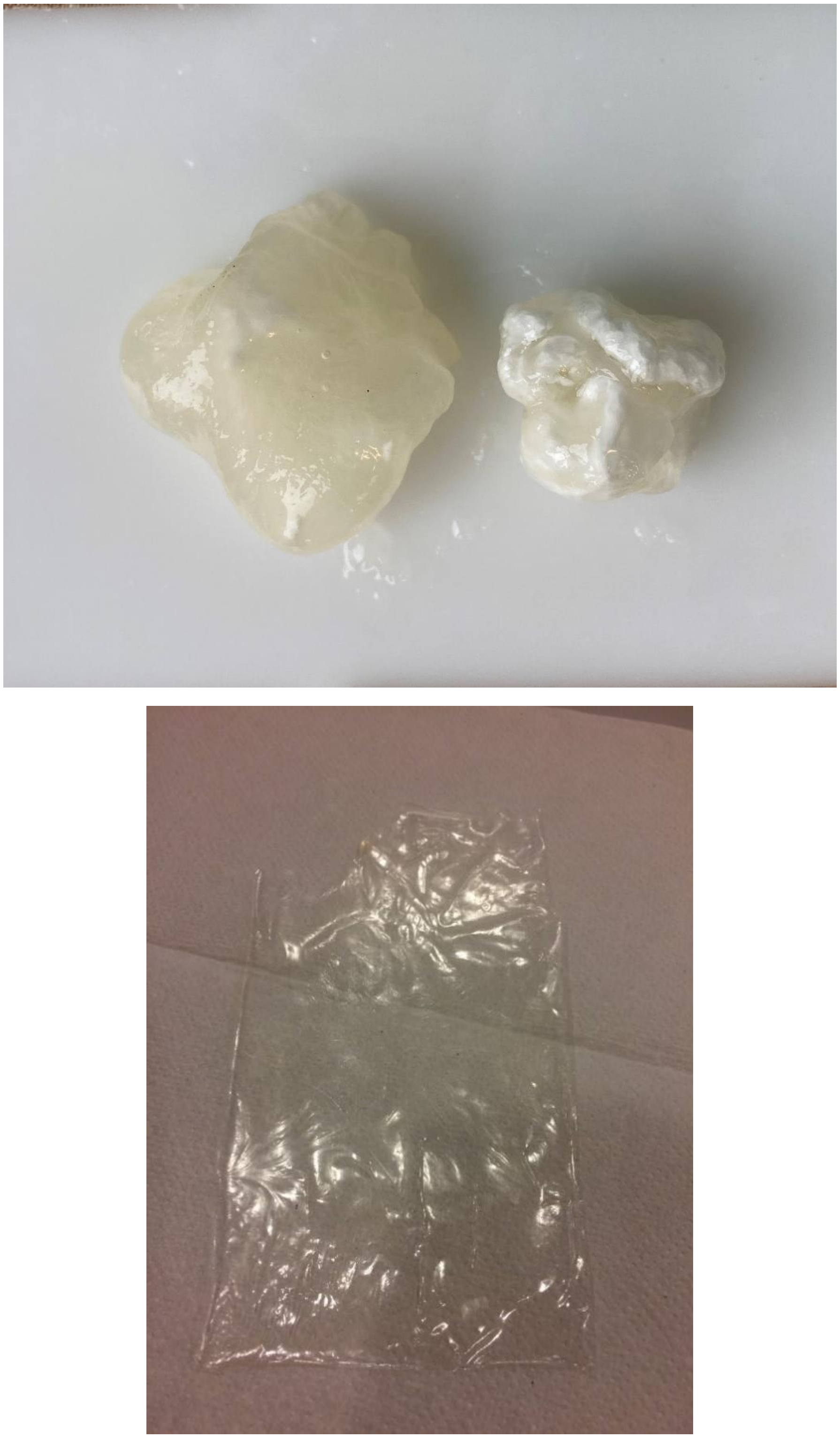

Figure S9. Photograph of PAA-PDADMA complex after cleaning, and saloplastic in the dry state (storage condition)

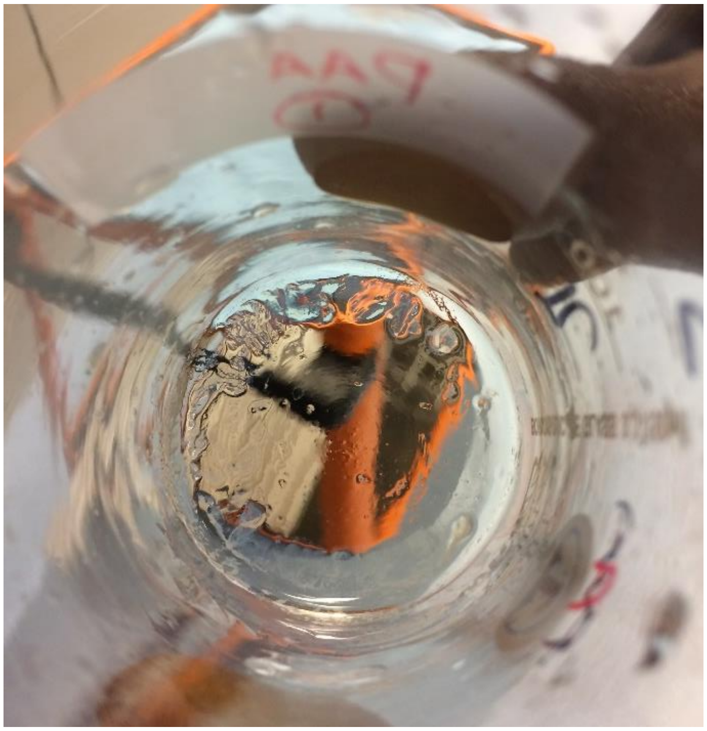

Figure S10. PAA-PDADMA saloplastic weakened and became gel-like in 1M NaCl or 0.1M HCl for 24h.

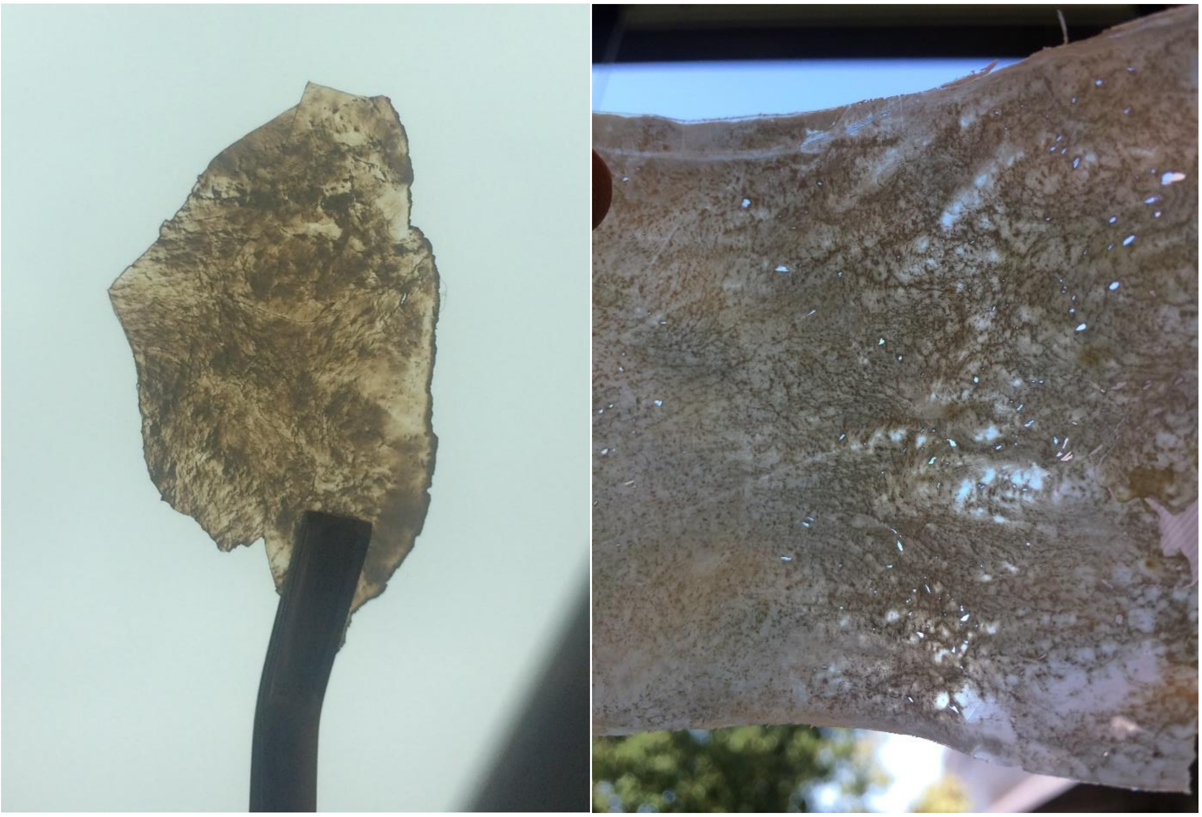

Figure S11: PSS-PEI saloplastic hot-pressed at 70°C with inhomogeneities, non-plasticized patches, and holes

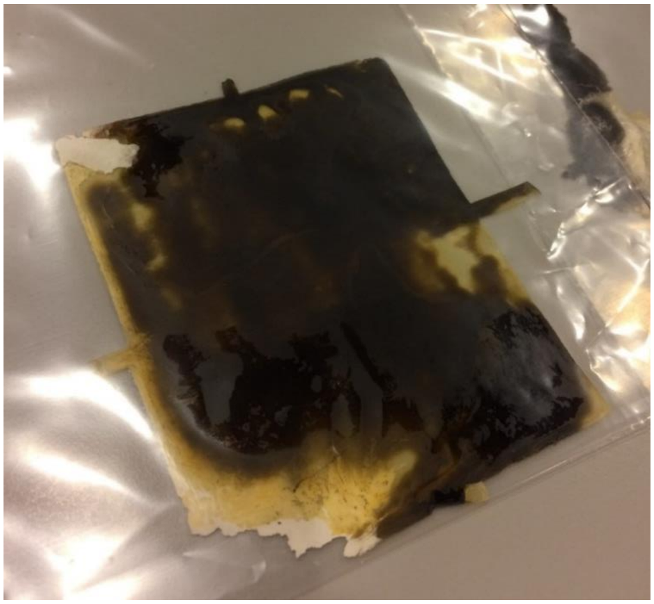

Figure S12: PSS-PEI saloplastic hot-pressed at 90°C seems charred

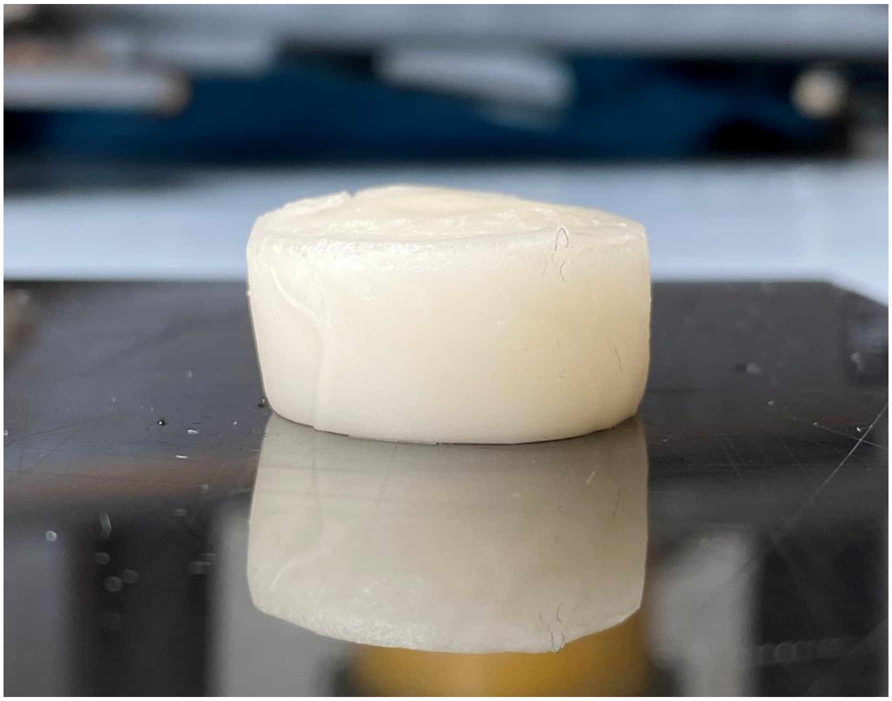

Figure S13: PAA-PAH complex after centrifugation and allowed to concentrate by drying

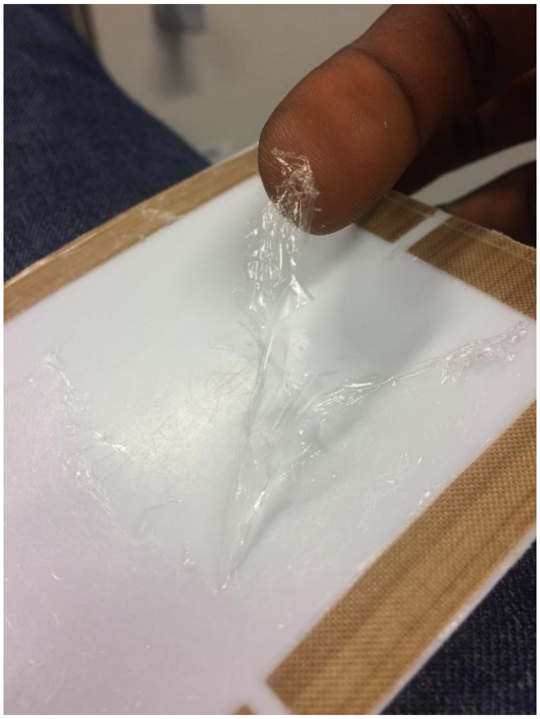

Figure S14: PAA-PAH hot pressed saloplastic unstable in storage conditions (humidity); inconvenient to handle

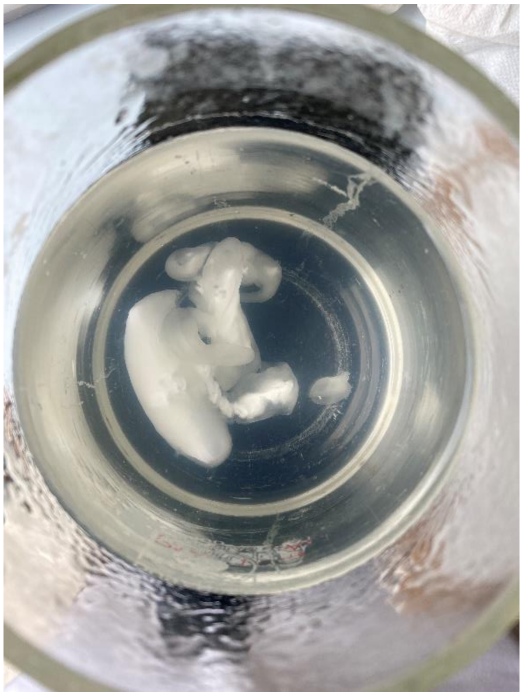

Figure S15: Typical best-complex formed by a combination of two weak polyelectrolytes

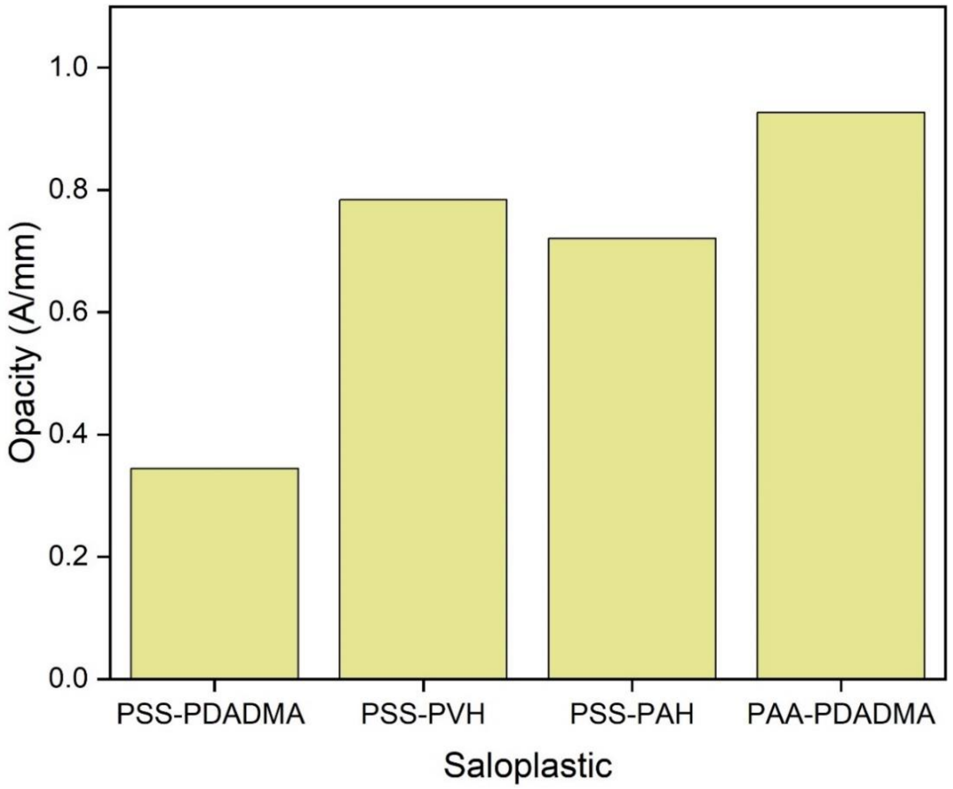

Figure S16. Opacity of different saloplastics measured using UV-VIS spectroscopy.

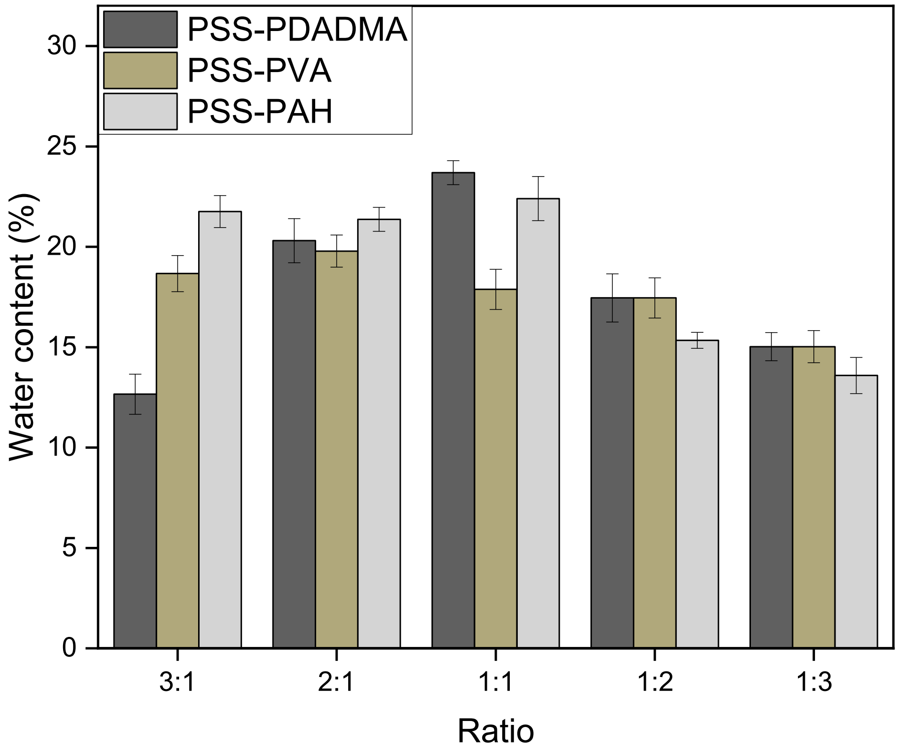

Figure S17. Water content of saloplastics made from different polyelectrolyte combinations and ratios. Error bars represent an average of at least 3 measurements

PAA-PDADMA swells extensively, with a water uptake of 62.4 ± 9.0 %, unlike the three combinations studied in figure SI 17.

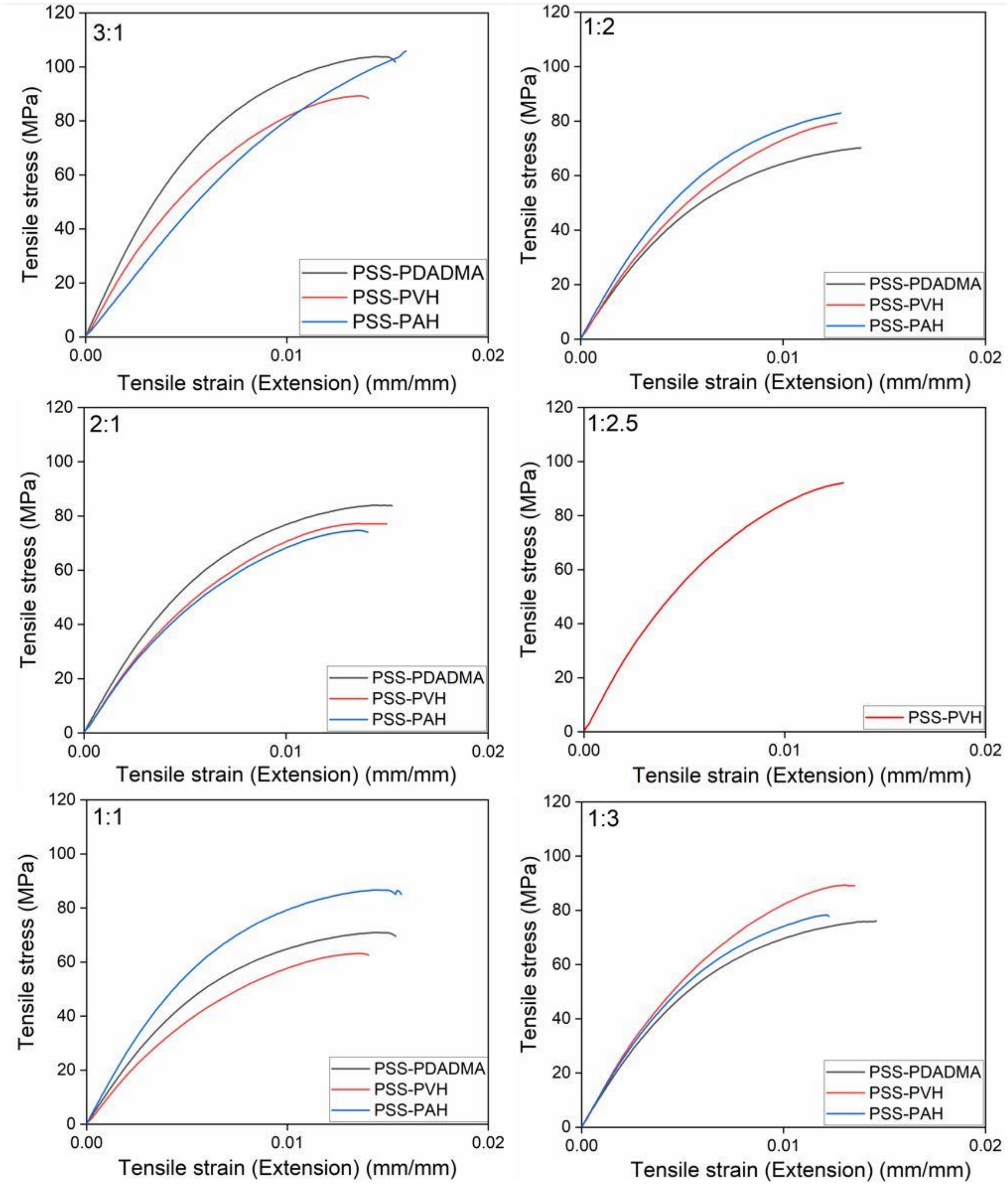

Figure S18. Representative stress-strain curves for the values described in Figure 3 of the manuscript. Each graph represents a specific ratio mentioned in the top-left corner of the respective graph. All curves are single measurements.

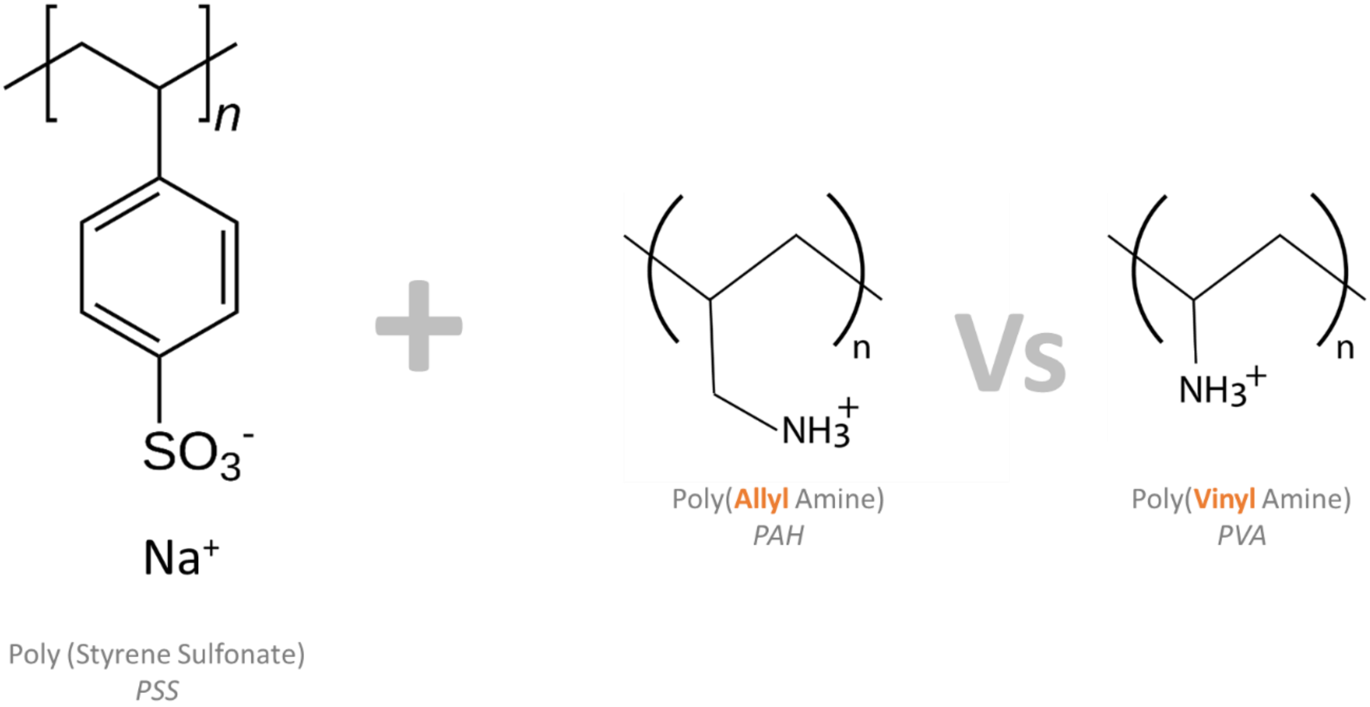

Figure S19. Line diagrams of Na-PSS with PAH and PVA showing difference in structures of PAH and PVA.

Ratio	3:1	2:1	1:1	1:2	1:2.5	1:3
**PSS-PDADMA**	5922 ± 339	5739 ± 299	5211 ± 268	5430 ± 174	NA	5784 ± 218
**PSS- PVH**	6205 ± 184	5894 ± 258	5002 ± 225	6011 ± 215	6424 ± 276	6516 ± 191
**PSS-PAH**	6395 ± 226	5813 ± 169	5814 ± 96	6096 ± 244	NA	6239 ± 152
**PSS-PAA**	NA	NA	3116 ± 374	NA	NA	NA

Table S2. Tensile strengths of saloplastics in megapascals (MPa). Each value is an average of at least 3 measurements measured at a humidity of 42%. NA= Not Applicable.

Ratio	3:1	2:1	1:1	1:2	1:2.5	1:3
**PSS-PDADMA**	-0.31 ± 0.15	0.45 ± 0.16	1.01 ± 0.15	0.96 ± 0.14	NA	1.11 ± 0.31
**PSS- PVH**	0.35 ± 0.17	0.38± 0.2	0.13 ± 0.09	-0.61 ± 0.15	-1.08 ± 0.18	-0.51 ± 0.32
**PSS-PAH**	-0.7 ± 0.32	-0.3 ± 0.35	0.37 ± 0.19	0.98 ± 0.19	NA	1.02 ± 0.15
**PSS-PAA**	NA	NA	0.68 ± 0.36	NA	NA	NA

Table S3. Net Ion exchange capacities of each in mmol g^{-1}. Each value is an average of at least 2 measurements. NA= Not Applicable. The negative sign is symbolic of the negative charge of the membrane, used to distinguish between anion and cation exchange membranes respectively.

Ratio	3:1	2:1	1:1	1:2	1:2.5	1:3
**PSS-PDADMA**	3.47 ± 0.29	3.46 ± 0.18	2.29 ± 0.18	3.19 ± 0.12	NA	2.2 ± 0.26
**PSS- PVH**	4.10 ± 0.41	4.34 ± 0.32	3.91 ± 0.47	3.40 ± 0.40	2.75 ± 0.16	4.02 ± 0.33
**PSS-PAH**	4.25 ± 0.37	4.94 ± 0.50	5.11 ± 0.43	3.39 ± 0.39	NA	2.81 ± 0.25
**PSS-PAA**	NA	NA	4.70 ± 0.36	NA	NA	NA

Table S4. Resistance offered to the passage of counterions by each membrane after subtracting the solution resistance normalized with area. All values are presented in $\Omega.cm^2$. Each value is an average of at least 3 measurements. NA= Not Applicable.

Ratio	3:1	2:1	1:1	1:2	1:2.5	1:3
**PSS-PDADMA**	-25 ± 1.92	24 ± 2.45	89 ± 1.9	71 ± 2.11	NA	90 ± 2.82
**PSS- PVH**	37 ± 1.92	8 ± 0.99	-37 ± 1.77	-66 ± 1.96	-88 ± 1.05	-12 ± 1.36
**PSS-PAH**	-52 ± 1.77	8 ± 1.05	26 ± 1.08	93 ± 0.63	NA	95 ± 0.94
**PSS-PAA**	NA	NA	-58 ± 5.13	NA	NA	NA

Table S5. Permselectivities of each membrane in %. Each value is an average of at least 3 measurements. NA= Not Applicable. The negative sign is symbolic of the negative charge of the membrane, used to distinguish between anion and cation exchange membranes respectively.

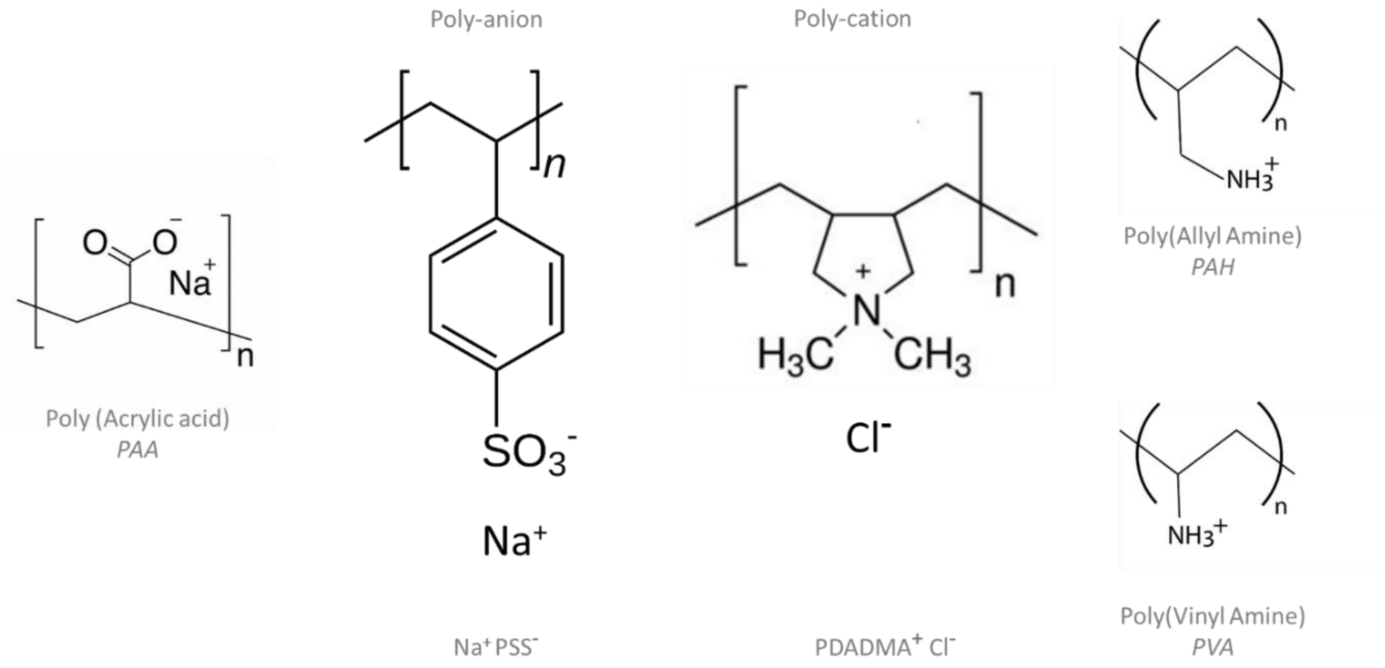

Figure S20: Polycations and polyanions that successfully led to processible plastics from their complexes.

Footnotes

Stability after staying in acids and bases for 14 days was calculated by measuring permselectivities before and after the storage. While PSS-PDADMA Note that the experiments themselves were not conducted in high or low pH.

PAH and PVH differences: The polyvinylamine hydrochloride and polyallylamine hydrochloride that were used had the same counterion, chloride, and have similar structures except for the presence of an excess carbon and two associated hydrogens in the latter. However, their individual complexes with polystyrene sulfonate (PSS) were found to be positively and negatively charged respectively. The nature of charge is maintained on processing into saloplastics, confirmed by permselectivity measurements and ion exchange capacity as shown in their respective sections.

Summary	Best ratio	Thickness	Young's modulus	Water Uptake	Ion exchange capacity	Charge density	Area resistance	Permselectivity (AEM/CEM) (0.03/0.15 M KCl)
		μm	MPa	%	mmol g^{-1}	mmol g^{-1}	$\Omega \cdot cm^2$	%
**PAA-PDADMA**	1:1	97 ± 11	3100 ± 380	63 ± 9	0.7 ± 0.4	0.43	4.70 ± 0.4	58 ± 5 (CEM)
**Commercial membranes**								
**Neosepta AMX**	NA	141 ± 6	3900 ± 190	19 ± 2	1.6 ± 0.2	1.34	2.40	94 (AEM)
**Neosepta ACS**	NA	127 ± 3	3600 ± 220	31 ± 3	1.7 ± 0.3	1.30	3.80	95 (AEM)
**Neosepta CMX**	NA	170 ± 9	4300 ± 160	24 ± 1	1.5 ± 0.3	1.21	3.00	97 (CEM)
**Selemion CMV**	NA	120 ± 3	1900 ± 310	32 ± 4	1.6 ± 0.2	1.21	3.60	95 (CEM)

Table S6. PAA-PDADMA membrane properties compared to commonly used commercial membranes. All the measurements, including those of the commercial membranes, were performed in our labs. Error bars indicate an average of at least two measurements, and more if a significant variation was observed.

PAA-PDADMA plastics were stable between pH 5 and 9 owing to the pKa value of PAA being 4 below which its carboxylic acid groups get protonated.[7,8]

System	Ratio	3:1	2:1	1:1	1:2	1:3
**1**	PSS-PDADMA	2-9	1-12	1-14	1-14	1-12
**2**	PSS- PVH	1-8	1-8	1-9	1-9	2-9
**3**	PSS-PAH	2-8	1-8	1-9	1-9	2-8
**4**	PAA-PDADMA	NA	NA	5-9	NA	NA

Table S7. pH values indicating the stability of PAA-PDADMA hot-pressed saloplastics compared to other saloplastics

References for Supplementary Information

(1) Krishna B, A.; Lindhoud, S.; de Vos, W. M. Hot-Pressed Polyelectrolyte Complexes as Novel Alkaline Stable Monovalent-Ion Selective Anion Exchange Membranes. *J. Colloid Interface Sci.* **2021**, *593*, 11–20. https://doi.org/10.1016/j.jcis.2021.02.077.

(2) Krishna B, A.; Zwijnenberg, H. J.; Lindhoud, S.; de Vos, W. M. Sustainable K+/Na+ Monovalent-Selective Membranes with Hot-Pressed PSS-PVA Saloplastics. *J. Memb. Sci.* **2022**, *652*, 120463. https://doi.org/10.1016/j.memsci.2022.120463.

(3) Shamoun, R. F.; Reisch, A.; Schlenoff, J. B. Extruded Saloplastic Polyelectrolyte Complexes. *Adv. Funct. Mater.* **2012**, *22* (9), 1923–1931. https://doi.org/10.1002/adfm.201102787.

(4) Fu, J.; Fares, H. M.; Schlenoff, J. B. Ion-Pairing Strength in Polyelectrolyte Complexes. *Macromolecules* **2017**, *50* (3), 1066–1074. https://doi.org/10.1021/acs.macromol.6b02445.

(5) Lai, X.; Gao, G.; Watanabe, J.; Liu, H.; Shen, H. Hydrophilic Polyelectrolyte Multilayers Improve the ELISA System: Antibody Enrichment and Blocking Free. *Polymers (Basel).* **2017**, *9* (2), 51. https://doi.org/10.3390/polym9020051.

(6) Fu, J.; Fares, H. M.; Schlenoff, J. B. Ion-Pairing Strength in Polyelectrolyte Complexes. *Macromolecules* **2017**, *50* (3), 1066–1074. https://doi.org/10.1021/acs.macromol.6b02445.

(7) Schilli, C. M.; Zhang, M.; Rizzardo, E.; Thang, S. H.; Chong, Y. K.; Edwards, K.; Karlsson, G.; Müller, A. H. E. A New Double-Responsive Block Copolymer Synthesized via RAFT Polymerization: Poly(N-Isopropylacrylamide)-Block-Poly(Acrylic Acid). *Macromolecules* **2004**, *37* (21), 7861–7866. https://doi.org/10.1021/ma035838w.

(8) Khanlari, S.; Dubé, M. A. Effect of PH on Poly(Acrylic Acid) Solution Polymerization. *J. Macromol. Sci. Part A Pure Appl. Chem.* **2015**, *52* (8), 587–592. https://doi.org/10.1080/10601325.2015.1050628.
